# Transitions between Andean and Amazonian centers of endemism in the radiation of some arboreal rodents

**DOI:** 10.1186/1471-2148-13-191

**Published:** 2013-09-09

**Authors:** Nathan S Upham, Reed Ojala-Barbour, Jorge Brito M, Paúl M Velazco, Bruce D Patterson

**Affiliations:** 1 Committee on Evolutionary Biology, University of Chicago, Chicago, IL 60637, USA; 2 Center for Integrative Research, Field Museum of Natural History, Chicago, IL 60605, USA; 3 Departamento de Ciencias Biológicas, Escuela Politécnica Nacional, Quito, Ecuador; 4 Department of Mammalogy, American Museum of Natural History, New York, NY 10024, USA

**Keywords:** Biogeography, *Ex situ* diversification, Molecular phylogeny, Divergence timing, South America, Andes, Amazonia, Neotropics, Rodentia, Echimyidae

## Abstract

**Background:**

The tropical Andes and Amazon are among the richest regions of endemism for mammals, and each has given rise to extensive *in situ* radiations. Various animal lineages have radiated *ex situ* after colonizing one of these regions from the other: Amazonian clades of dendrobatid frogs and passerine birds may have Andean ancestry, and transitions from the Amazon to Andes may be even more common. To examine biogeographic transitions between these regions, we investigated the evolutionary history of three clades of rodents in the family Echimyidae: bamboo rats (*Dactylomys-Olallamys-Kannabateomys*), spiny tree-rats (*Mesomys-Lonchothrix*), and brush-tailed rats (*Isothrix*). Each clade is distributed in both the Andes and Amazonia, and is more diverse in the lowlands. We used two mitochondrial (cyt**-***b* and 12S) and three nuclear (GHR, vWF, and RAG1) markers to reconstruct their phylogenetic relationships. Tree topologies and ancestral geographic ranges were then used to determine whether Andean forms were basal to or derived from lowland radiations.

**Results:**

Four biogeographic transitions are identified among the generic radiations. The bamboo rat clade unambiguously originated in the Amazon *ca.* 9 Ma, followed by either one early transition to the Andes (*Olallamys*) and a later move to the Amazon (*Dactylomys*), or two later shifts to the Andes (one in each genus). The Andean species of both *Dactylomys* and *Isothrix* are sister to their lowland species, raising the possibility that highland forms colonized the Amazon Basin. However, uncertainty in their reconstructed ancestral ranges obscures the origin of these transitions. The lone Andean species of *Mesomys* is confidently nested within the lowland radiation, thereby indicating an Amazon-to-Andes transition *ca.* 2 Ma.

**Conclusions:**

Differences in the timing of these biogeographic transitions do not appear to explain the different polarities of these trees. Instead, even within the radiation of a single family, both Andean and Amazonian centers of endemism appear enriched by lineages that originated in the other region. Our survey of other South American lineages suggests a pattern of reciprocal exchange between these regions—among mammals, birds, amphibians, and insects we found no fewer than 87 transitions between the Andes and Amazon from Miocene-Pleistocene. Because no clear trend emerges between the timing and polarity of transitions, or in their relative frequency, we suggest that reciprocal exchange between tropical highland and lowland faunas in South America has been a continual process since *ca.* 12 Ma.

## Background

The tropical region that extends from Southern Mexico and the Antilles to Paraguay and Northern Argentina is home to some of the world’s richest biotas. On a global scale, nearly 40% of all bird species are found in the American tropics [1], as are a quarter of all mammal species [2]. Species density maps for terrestrial vertebrates [3-5] show that extremely rich faunas blanket most of the tropical Andes, Amazonia, the Guianan Shield, and Atlantic Forest subregions. Each subregion harbors distinctive and historically differentiated faunas [2,6], which makes beta diversity (species turnover relative to distance) a sizeable portion of the regional total. Nevertheless, alpha diversities (species richness) are greatest along the Andean-Amazonian interface at ~1500 m [3,7,8]. Nature reserves that straddle this interface contain up to 8% of the world’s avifauna and at least 226 species of mammals [1,9,10].

Where, when, and how did this diversity of species originate? Because species richness peaks at the interface of Andean and Amazonian subregions, both areas are implicated. Both have also had dynamic geohistories. Although precursors of the Andean Cordillera are ancient, the first major period of orogenic uplift and growth was triggered ~12-10 Ma in the central portion [11]. This late Neogene event created a succession of newly emergent habitats in the Central and Northern Andes, prompting many to argue that Andean endemics were derived from Amazonian ancestors that colonized novel biomes (e.g., birds and butterflies) [12-15]. On the other hand, the Pebas wetland system encompassed much of Western Amazonia until the late Miocene [16-18], so this area might only have been colonized by terrestrial organisms thereafter. Thus, others argue that some Amazonian radiations have Andean roots (e.g., amphibians and mammals) [19-21]. Undoubtedly, the individual ecologies, biogeographic histories, and chance events associated with groups distributed in and across these two regions have influenced their patterns of diversification [22-24]. Yet it remains unclear whether the timing and polarity of diversification across the Andes-Amazon transition are consistently related. Did the uplift of the Andes and resultant draining of the Pebas wetlands in the Miocene and Pliocene alter the role of Andean habitats in Amazonian diversification?

**Figure 1:**
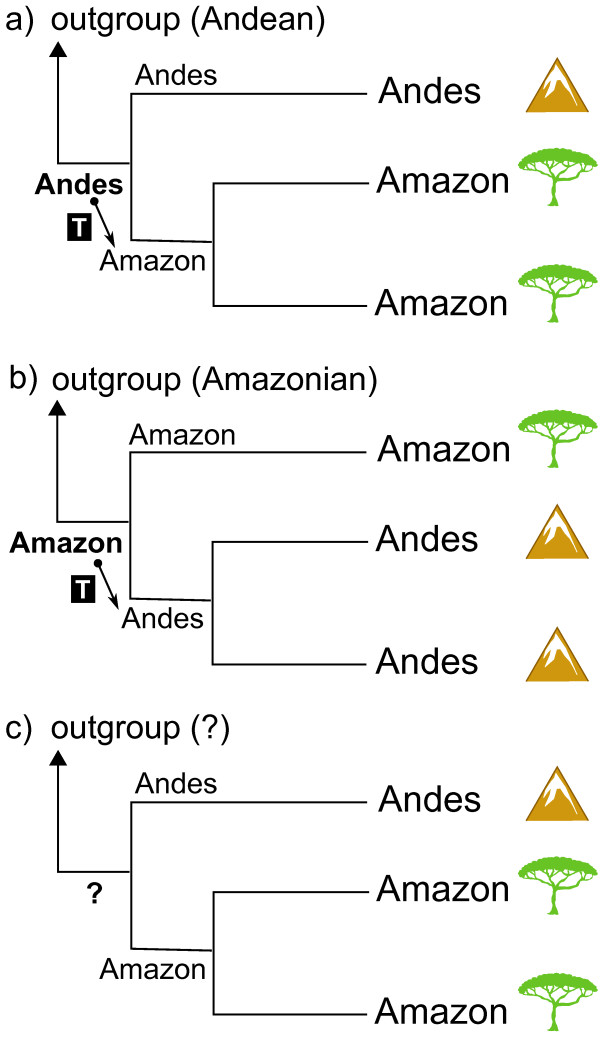
**Most parsimonious interpretations of phylogenetic hypotheses to identify the geographic origin of species.** Shown are possible derivations of species from **(a)** Andean or **(b)** Amazonian ancestors. Determining the polarity of a biogeographic transition (T, inside box) requires a phylogeny with at least three in-group members and a well-supported outgroup rooting the tree. **(c)** If the outgroup is unknown or poorly supported, it is not possible to identify the geographic range of the stem ancestor from which the transition originated. Using Bayesian or maximum-likelihood frameworks for reconstructing ancestral ranges (e.g., in Lagrange [25]) incorporates this topological data along with information such as the distribution of branch lengths and the historical connectivity of regions.

To investigate questions of biogeographic polarity in a phylogenetic framework, a rooted tree with at least three in-group members is required (Figure 1). Here we employ phylogenetic hypotheses to identify the derivation of species from Andean (Figure 1a) or Amazonian (Figure 1b) ancestors, focusing on modern clades of rodents of the family Echimyidae (Caviomorpha: Octodontoidea) that are co-distributed among these regions. Our goals are to (i) confirm the monophyly of these co-distributed clades, (ii) calibrate the timing of molecular divergences using fossil ages, (iii) reconstruct ancestral biogeographic ranges and the polarity of biogeographic transitions, and, (iv) compare the resulting patterns of Andes-Amazon exchange to other Neogene radiations of mammals, birds, amphibians, and insects.

### Study organisms

Commonly called “spiny rats,” the Echimyidae represents the most speciose group of caviomorph rodents (guinea pigs and their allies). The family includes 91 extant species in 22 genera, excluding a number of poorly understood Caribbean taxa (either allied with Echimyidae or Capromyidae). All echimyids are endemic to Central and South America, as are a host of successive sister groups Capromyidae, Octodontidae + Ctenomyidae, Abrocomidae, Chinchilloidea, and Cavioidea + Erethizontoidea [26,27]. Echimyids occupy a wide range of habitats, from grasslands to restinga, caatinga, cerrado, cloud forests, and lowland and montane rainforests. Their radiation apparently began in the Early Miocene (23–16 Ma), with the crown divergence of Eastern Brazilian and the arboreal + terrestrial clades [26]. The subsequent rapid diversification of echimyids, particularly among arboreal species, may explain why basal nodes in the phylogeny have been difficult to resolve [28-30].

**Figure 2:**
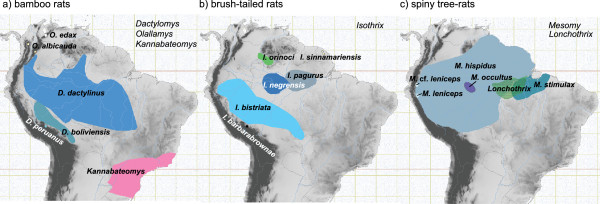
**Geographic ranges of target clades examined in this study (Andean species are noted in parentheses). (a)** bamboo rats (*Dactylomys peruanus*, *Olallamys albicauda,* and *O. edax*); **(b)** brush-tailed rats (*Isothrix barbarabrownae*); and **(c)** spiny tree-rats (*Mesomys* cf. *leniceps*). Range maps are from the IUCN [32] database and are overlaid on a digital elevation map of northern South America (progressively darker shades of gray represent elevations from sea level to 6900 m).

Although systematic relationships of Echimyidae have not been thoroughly sampled with molecular data, the arboreal taxa appear to form a clade that includes *Dactylomys, Echimys*, *Isothrix*, *Kannabateomys, Lonchothrix*, *Makalata, Mesomys, Phyllomys*, and *Toromys*[27]. Current taxonomy [31] implies that a number of unsampled genera probably belong to the same clade, including *Callistomys*, *Diplomys, Olallamys*, *Pattonomys,* and *Santamartamys*. Among arboreal genera sampled for DNA, three genera are represented by at least three species and have distributions both in Amazonia and Andean montane and/or cloud forests. Each genus contains a predominantly lowland Amazonian radiation and one or more highland Andean species (Figure 2).

### Bamboo rats (*Dactylomys* and allies)

The genus *Dactylomys* Geoffroy Saint-Hilaire, 1838 [33] includes large arboreal rodents with blocky heads, coarse (not spiny) fur, nails instead of claws on the feet, and a long, naked, scaly tail furred only at the base. They have an especially broad, heavy dentition to consume a predominantly folivorous diet that includes bamboo leaves and shoots [34]. Their characteristic appearance led to their long-standing recognition as a distinct subfamily of Echimyidae, the Dactylomyinae [35]. Molecular analyses indicate this genus is sister to *Kannabateomys*, a similar bamboo rat from the Atlantic Forests of Eastern Brazil [27,28]. A third member in this group, *Olallamys*, is known from two species in the Northern Andes of Colombia and Venezuela (*albicauda* and *edax*), but neither has yet been included in molecular phylogenetic analyses.

*Dactylomys* is thought to include three species (Figure 2a), one of which is Andean: *boliviensis* from lowland tropical forests in southeastern Peru and Bolivia; *dactylinus* from lowland tropical forests through most of Amazonia from Colombia to Bolivia, from its mouth to foothills; and *peruanus*, known only from Andean cloud forests at 1000–3000 m elevations in southeastern Peru [36]. Patterson and Velazco [37] showed that the Andean species is sister to the remaining two (*peruanus* (*boliviensis + dactylinus*)); without a sister group in Amazonia or the Andes, they had no means to establish the polarity of this bifurcation.

### Brush-tailed rats (*Isothrix*)

The genus *Isothrix* Wagner, 1845 [38] includes several squirrel-sized arboreal rats with very dense, soft fur and long bushy tails. It is securely placed as a member of the arboreal clade [27,28], but seems distantly related to other arboreal taxa. A number of other soft-furred arboreal rats classified in Echimyinae (i.e., *Callistomys, Diplomys,* and *Santamartamys*) have never been included in molecular analyses, so it is possible that one of these is closer to *Isothrix* than any sampled taxon cf. [31].

*Isothrix* is thought to contain six species (Figure 2b): one Andean and the remainder found in moist broadleaf forests. The recently described species *barbarbrownae* is known only from cloudforests at 1800–2000 m in southeastern Peru; *bistriata* from lowland rainforests in Western Amazonia; *negrensis* from Central Amazonia near the mouth of the Rio Negro; *orinocensis* from the Orinoco drainage; *pagurus* from the lower Amazon; and *sinnamariensis* from the Atlantic drainage in the Guianas [36,39]. Although their phylogenetic relationships are not fully resolved [37], the Andean species is known to be sister to a group containing a near-polytomy: (*barbarabrownae* ((*bistriata*)(*pagurus + sinnamariensis*)(*negrensis + orinocensis*)). Without a known sister group, this group could have originated in the Andes or Amazon [see also [40].

### Spiny tree-rats (*Mesomys* and *Lonchothrix*)

The genus *Mesomys* Wagner, 1845 [38] includes several small, heavily-spined arboreal rats with characteristically short broad feet and sharp claws, as well as sparsely haired tails ending in a distinct tuft. It is sister to the monotypic genus *Lonchothrix* of the lower Amazon Basin [28,29], but the relationships of this clade to other arboreal echimyids remain unclear.

*Mesomys* includes at least three [19,31,41] or four [36] species, one of which is Andean. These are: *hispidus*, a widespread and highly variable form distributed over most of Amazonia west of the Rio Tapajós cf. [42]; *leniceps*, from Andean cloud forests in northern Peru; *occultus*, from central Amazonia; and *stimulax*, east of the Tapajós and south of the Amazon in Brazil (Figure 2c). However, the monophyly of the genus has never been thoroughly tested. Establishing monophyly is a non-trivial issue for *Mesomys*, as Tate’s [43] nomenclatural synopsis documented its historical confusion with *Makalata*, *Echimys*, *Phyllomys*, *Clyomys*, and *Euryzygomatomys*. Patton et al. [19] established the current phylogenetic framework for this group. Analyzing three species over 798 bp of cyt-*b*, they recovered the grouping (*occultus* (*hispidus* + *stimulax*)). However, the omission of the Andean form *leniceps* renders these relationships mute on the biogeographic origins of this group.

## Methods

### Taxon and gene sampling

The arboreal clade of Echimyidae includes four lineages [26,27], which comprise our in-group for molecular analyses: *Isothrix*, *Mesomys* + *Lonchothrix*, *Toromys* + *Makalata* + *Phyllomys* + *Echimys*, and *Dactylomys* + *Kannabateomys* (and presumably *Olallamys*; Emmons 2005). We ensured that all in-group samples were vouchered by museum specimens to corroborate species identifications, and where possible employed multiple representatives for each species (Table 1; Additional file 1 lists all in-group localities). We sampled all known species for *Isothrix*, *Mesomys, Lonchothrix, Dactylomys*, and *Kannabateomys*, but were missing 1 *Olallamys* species, 1 *Toromys*, 2 *Makalata*, 11 *Phyllomys*, and 2 *Echimys*[31,36,44].

**Table 1:** Genetic sampling for this study, showing GenBank accession numbers for the five gene regions examined

**Species**	**Collector #**	**Museum voucher**	**Gene region**				
			**cyt-*b***	**12S rRNA**	**GHR**	**vWF**	**RAG1**
Chinchilloidea							
*Chinchilla lanigera*		(FMNH 178049)	AF464760	AF520696	AF332036	AJ238385	**KF590658**
Octodontoidea							
Abrocomidae							
*Abrocoma bennettii*			AF244387		FJ855213	AJ251143	JN633625
*Abrocoma cinerea*			AF244388	AF520666	AF520643		
Octodontidae							
*Octodontomys gliroides*		(FMNH 162890)	AF370706	AF520683	AF520649	**KF590672**	**KF590663**
Ctenomyidae							
*Ctenomys coyhaiquensis*		(FMNH 134300)	AF119112	**KF590700**	**KF590678**	**KF590666**	**KF590659**
Capromyidae							
*Capromys pilorides*			AF422915	AF433926	AF433950	AJ251142	JN633628
Echimyidae							
*Trinomys iheringi*		(FMNH 141667)	EU313254	AF422868	**KF590695**	**KF590677**	EU313337
*Thrichomys apereoides*			EU313252	AF422855	JX515325	AJ849315	EU313334
*Myocastor coypus*			EU544663	AF520669	AF520662	AJ251140	AY011892
*Proechimys cuvieri*		(FMNH 175256)	AJ251400	**KF590707**	**KF590693**	**KF590675**	**KF590665**
**In-groups**							
*Makalata macrura*	JLP 7197	MVZ 153637	EU313236		**KF590687**		EU313325
*Makalata macrura*	JLP 15214	MVZ 194324	L23356	AF422879		AJ849312	EU313328
*Toromys grandis*	AMO 824	FMNH 92198	**KF590699**		**KF590694**	**KF590676**	EU313336
*Phyllomys blainvillii*	LPC 246	MVZ 197568	JF297836	**KF590706**	**KF590692**	JF297734	**KF590664**
*Phyllomys blainvillii*	LMP 27	MNRJ 43810	U35412	AF422876	JX515331	JF297732	JX515323
*Echimys chrysurus*	LHE 555	USNM 549594	L23341	AF422877	JX515333		
*Echimys chrysurus*		ROM 111578	EU313213				EU313303
*Lonchothrix emiliae*		INPA 2472	AF422921	AF422857			
*Mesomys occultus*	JUR 501	MVZ 194396	L23388	AF422858	**KF590689**		EU313331
*Mesomys occultus*	MNFS 201		U35415				
*Mesomys stimulax*	MDC 550	USNM 549807	L23389				
*Mesomys stimulax*	LHE 572	USNM 549808	L23392				
*Mesomys* cf*. leniceps*	JBM 368	MEPN 12212	**KF590705**	**KF590696**	**KF590688**	**KF590671**	**KF590662**
*Mesomys hispidus*	MNFS 436	MVZ 194378	L23385	AF422860		AJ849305	
*Mesomys hispidus*	MNFS 745	MVZ 194391	L23395	AF422861			EU313322
*Mesomys hispidus*	LHE 748		L23396				
*Mesomys hispidus*	LHE 836	USNM 579619	L23393				
*Mesomys hispidus*	MNFS 909	MVZ 194393	L23398				
*Mesomys hispidus*	ALG 14162	MBUCV	L23371				
*Dactylomys boliviensis*	MNFS 988	MVZ 194298	L23339	AF422875	JX515334	AJ849307	
*Dactylomys boliviensis*	BDP 3942	FMNH 175249	EU313204		**KF590679**		EU313298
*Dactylomys boliviensis*	SS 2225	FMNH 175250	EU313205		**KF590680**		EU313299
*Dactylomys dactylinus*		INPA 2477	L23335	AF422874			
*Dactylomys dactylinus*	LHE 607	USNM 549842	L23336				EU313301
*Dactylomys dactylinus*	LHE 878	USNM 579620	L23337		**KF590681**	**KF590667**	EU313300
*Dactylomys peruanus*	LHE 1398	USNM 582148	EU313207				
*Dactylomys peruanus*	LHE 1374	MUSM13052	EU313206				
*Kannabateomys amblyonyx*	YL 182		AF422916	AF422849			
*Kannabateomys amblyonyx*	CTX 2942		AF422917	AF422850		AJ849310	
*Olallamys albicauda*	PH 6445	FMNH 71128	**KF590697**		**KF590690**	**KF590673**	
*Olallamys albicauda*	PH 6488	FMNH 71129	**KF590698**		**KF590691**	**KF590674**	
*Isothrix barbarabrownae*	BDP 3878	MUSM 16819	EU313214	**KF590701**	**KF590682**	**KF590668**	EU313304
*Isothrix bistriata*	MNFS 471	MVZ 194315	L23349		JX515336	AJ849308	
*Isothrix bistriata*	RSV 2293	MUSM 13305	EU313217				EU313307
*Isothrix negrensis*	MNFS 97	INPA	L23355	AF422873			
*Isothrix negrensis*	JLP 16749	INPA	EU313220				
*Isothrix orinoci*		USNM 406370	EU313223	**KF590702**	**KF590683**	**KF590669**	**KF590660**
*Isothrix orinoci*		USNM 415193	EU313225				
*Isothrix pagurus*	LHE 141	USNM 555639	EU313227	**KF590703**	**KF590684**	**KF590670**	**KF590661**
*Isothrix pagurus*		INPA 2463	L23348				
*Isothrix sinnamariensis*		ROM 106624	AY745734	**KF590704**	**KF590685**		EU313312
*Isothrix sinnamariensis*		T4377	EU313228		**KF590686**		EU313313

Sequences in bold (with lengths in base pairs, bp) were newly generated for this study. Species listed without museum or collector numbers are chimeric assemblies; chimeras with newly generated sequence data have museum numbers in parentheses that correspond to voucher specimens.

To root all trees, we included successive sister groups to the arboreal clade identified in previous analyses (Table 1) [26,27]. The designated outgroup was always *Chinchilla lanigera* from the superfamily Chinchilloidea, the sister group to all other sampled taxa in the superfamily Octodontoidea [27]. We generated an array of new DNA sequences for two mitochondrial (mtDNA) genes—cytochrome-*b* (cyt-*b*) and 12S ribosomal RNA (12S rRNA)—and three unlinked nuclear exons—growth hormone receptor exon 10 (GHR), von Willebrand factor exon 28 (vWF), and recombination activating gene 1 (RAG1; Table 1). These genes were selected on the basis of: (1) variation in evolutionary rates (mitochondrial vs. nuclear); (2) the diversity of taxa previously sampled; and (3) their demonstrated utility in caviomorph phylogenetics [e.g., [27,28,37].

**Table 2:** Nodal support values for single genes and combined gene data sets

	**Combined data sets**				**Single gene data sets**				
	**5-gene**		**2-gene**	**3-gene**					
**Node**	**Bayesian PP**	**ML bootstrap**	**mtDNA**	**nuclear exons**	**cyt-*b***	**12S rRNA**	**GHR**	**vWF**	**RAG1**
1	**1.00**	**100**	**100**	**100**	**100**	x	**100**	x	x
2	**0.89**	**82**	43	72	.	16	**88**	.	74
3	**1.00**	**99**	68	**99**	.	56	**94**	.	**91**
4	**1.00**	**100**	**79**	**100**	66	.	**100**	**100**	**99**
5	0.62	64	.	.	.	.	41	.	.
6	**1.00**	**90**	.	**88**	.	23	.	**88**	.
7	**1.00**	**96**	.	**92**	.	.	**77**	31	21
8	0.73	60	.	.	54	.	.	.	.
9	**1.00**	**94**	26	**77**	.	43	57	26	3
10	**1.00**	**83**	29	32	.	.	.	19	.
11	**1.00**	**100**	30	**91**	.	**95**	**77**	**81**	36
12	0.70	.	**76**	.	.	x	.	65	.
13	**1.00**	**100**	**100**	**100**	**100**	x	x	x	**100**
14	**0.99**	**86**	**81**	.	**83**	.	28	x	39
15	**1.00**	**100**	**100**	**98**	**100**	**100**	**86**	**100**	74
16	**1.00**	**100**	**100**	.	**100**	x	x	x	x
17	0.70	45	45	.	.	68	57	.	.
18	**1.00**	**100**	**100**	x	**91**	**100**	x	x	x
19	**1.00**	**100**	**100**	**98**	**99**	**84**	**100**	**100**	**76**
20	**1.00**	**100**	**100**	x	**100**	x	x	x	x
21	**1.00**	**98**	**97**	71	**92**	**95**	x	x	**81**
22	**1.00**	**100**	**100**	x	**99**	x	x	x	x
23	**0.90**	68	74	x	71	x	x	x	x
24	**0.98**	56	60	x	51	x	x	x	x
25	**0.97**	49	50	x	46	x	x	x	x
26	**1.00**	**88**	**92**	53	**91**	x	x	x	x
27	0.91	**81**	**79**	x	**76**	x	x	x	x
28	**1.00**	**90**	70	x	58	**84**	x	x	x
29	**1.00**	**100**	**96**	**95**	**91**	70	**100**	**90**	x
30	**1.00**	**100**	**100**	x	**100**	.	x	.	x
31	0.49	48	.	70	.	x	x	49	x
32	**1.00**	**100**	**100**	**100**	**100**	x	**100**	**79**	x
33	**1.00**	**100**	**100**	**100**	**100**	**100**	**100**	**100**	**100**
34	**1.00**	**100**	**100**	x	**100**	x	x	x	x
35	0.85	**85**	**79**	x	74	x	x	x	x
36	**1.00**	**100**	**100**	.	**100**	x	**77**	x	**85**
37	0.92	**85**	.	.	52	x	.	x	x
38	**1.00**	**97**	**97**	**82**	**98**	x	x	x	**99**
39	0.87	57	72	x	63	x	x	x	x
40	**1.00**	**100**	**100**	**100**	**98**	**100**	**100**	**97**	**97**
41	**1.00**	68	75	.	**91**	.	.	.	.
42	**1.00**	**100**	**100**	**95**	**99**	**100**	**98**	x	**76**
43	0.95	71	**79**	x	**82**	x	x	x	x
44	**1.00**	**86**	**89**	.	**89**	x	**94**	x	.
45	0.94	74	71	.	74	x	x	x	x
46	**1.00**	**100**	**100**	.	**100**	x	x	x	x
47	**1.00**	**100**	**100**	x	**99**	**99**	x	x	x
48	0.93	**84**	**90**	x	**91**	x	x	x	x
49	**1.00**	72	**77**	x	**77**	x	x	x	x

All values are maximum-likelihood (ML) bootstraps except for the Bayesian posterior probability (PP) listed for the 5-gene data set. Boldface values indicate ML bootstrap > 75 or a Bayesian PP > 0.95. Values denoted as “.” indicate a node not recovered despite all subtending taxa being sampled; those denoted “x” indicate an absent node due to unsampled taxa.

### DNA sequencing

We isolated genomic DNA from frozen fresh tissues (liver, kidney or muscle) preserved in ethanol, or from dried tissues (muscle, skin) adhering to museum voucher specimens. Fresh tissue DNA was extracted from 14 specimens using the DNeasy Blood & Tissue Kit (QIAGEN) and following the instructed protocol. All molecular laboratory work on fresh tissues was conducted in the Pritzker Laboratory for Molecular Systematics and Evolution (Field Museum of Natural History, Chicago, IL, USA) or in the Laboratório de Mastozoologia e Biogeografia (Universidade Federal do Espírito Santo, Vitória, ES, Brazil). Nucleic acid concentrations were quantified using a NanoDrop spectrophotometer (Thermo Fisher Scientific). Polymerase chain reaction (PCR) was carried out on DNA extractions to amplify target genes. Each PCR had a reaction volume of 10 μl and contained 1.0 μl of DNA template, 1.0 μl 10× reaction buffer, 1.0 μl of 8 mM premixed deoxynucleotide triphosphates (dNTPs; 200 μM each nucleotide in final reaction), 1.0 μl of 25 mM MgCl_2_, 0.5 μl of 10 mg/μl bovine serum albumin (Applied Biosystems), 4.4 μl of double-distilled H_2_O (dH_2_O), 0.1 μl of 5 U/μl AmpliTaq Gold™ DNA polymerase (Applied Biosystems), and 0.5 μl of each 10 μM priming oligonucleotide.

Dried tissue DNA, also called ancient DNA (aDNA), was extracted from six specimens. Extracts from three were available from a previous study [37], whereas three others were newly extracted and analyzed at the McMaster University Ancient DNA Centre (Hamilton, ON, Canada [45]). Prior DNA extracts were used to amplify additional genes from *Isothrix barbarabrownae* [Museo de Historia Natural, Universidad de San Marcos (MUSM) 16819, collected in 1999], *Isothrix orinoci* [US National Museum of Natural History (USNM) 406370, collected 1967], and *Isothrix pagurus* (USNM 555639, collected 1982). Newly analyzed were *Olallamys albicauda* [Field Museum of Natural History (FMNH) 71128, collected in 1956], *Olallamys albicauda* (FMNH 71129, collected 1956), and *Toromys grandis* (FMNH 92198, collected 1962). Dried tissues adhering to the cranium, mandible, and vertebrae (“crusties”) of these specimens were removed, shipped at ambient temperature to the Royal Ontario Museum (Toronto, Canada), and hand-carried to McMaster University. We used published aDNA protocols at McMaster, including the use of dedicated clean-room facilities for sample and buffer preparation, DNA extraction, PCR setup, and post-PCR work. We also used protective clothing and masks, and techniques to minimize contamination risk, such as UV light sterilization, PCR workstations, specifically designed primers, and filtered pipette tips [46,47]. Additional details of aDNA protocols, as well as the PCR primers, primer pairs, and sequencing protocols for all reactions are given in Additional file 2. Sequences were edited and assembled using Geneious 6.0.6 software (Biomatters). All new molecular sequences presented in this study have been deposited in GenBank (KF590658 – KF590707; Table 1).

### Gene alignment and combinability

Of the five genes analyzed, only cyt-*b* was sampled from every taxon. The other gene alignments contained varying amounts of unsampled or incompletely-sequenced genes (Table 1). Sequences from each gene were multiply aligned to establish character homology in relation to outgroups. For 12S rRNA, we aligned sequences based on the secondary structural model of Springer and Douzery [48]. At sites where multiple indels made sequence alignment ambiguous, we discarded a total of 127 base pairs (bp) from the initial alignment of 975 bp (positions 90–102, 126–131, 224–233, 292–297, 313–328, 381–388, 681–686, 745–767, 784–794, and 891–918). Protein-coding sequences were aligned using ClustalW 2.1 [49], and indels were verified to be in sets of three bp. Our resulting alignments were 1140 bp for cyt-*b*, 848 bp for 12S rRNA, 865 bp for GHR, 1263 bp for vWF, and 1102 bp for RAG1, for a total of 5218 bp of aligned mtDNA and nuclear exon sequence. The combined 5-gene alignment with partitions has been submitted to LabArchives and is available for download [50].

We paid extra attention to the cyt-*b* data set because this gene was sampled for all taxa, and is a useful indicator of mammal species relatedness [51]. Pairwise distances were calculated as mean distances among groups using uncorrected-p (raw number of nucleotide substitutions divided by length) and pairwise deletion of missing sites. A summary of this data is presented in Additional file 3. Since mutation saturation can also occur in cyt-*b* when synonymous substitutions occur in the third codon position, we plotted pairwise comparisons of overall percent sequence divergence versus number of substitutions. Without evidence of an asymptote as percent sequence divergence increased (Additional file 3), it was unnecessary to exclude any cyt-*b* data from the analyses.

Prior to combining gene alignments, we explored the possibility of incongruence between gene histories [52]. Maximum-likelihood (ML) phylogenetic trees were constucted for each gene using RAxML-HPC2 version 7.4.2 [53] on the XSEDE online computing cluster accessed via the CIPRES Science Gateway [54]. The best-fit model of nucleotide evolution for each gene was general time-reversible (GTR) plus among-site rate variation (Γ); some of the data sets also included the proportion of invariant sites (*I*) (data not shown--Akaike Information Criterion in MrModelTest 2.3) [55]. Concerns over the non-independence of *I* and Γ [53,56,57] motivated us to employ the simpler GTR+Γ model in all cases. Rapid bootstrapping was performed for each gene alignment using the “–f a” option and 1000 bootstrap replicates, resulting in best-scoring ML trees annotated with nodal support values. Identical analyses were also performed on mtDNA (cyt-*b* + 12S rRNA) and nuclear exon (GHR + vWF + RAG1) data sets to compare phylogenetic signal among genome sources. Node-by-node comparisons between all data sets found no major topological conflict (Table 2), allowing us to concatenate all five genes into a supermatrix of characters and thereby maximize both taxonomic and genetic diversity in the phylogeny approach reviewed by [58]. Presence of more than 2000 characters in the supermatrix was expected to override any statistical biases resulting from missing data [59,60].

### Phylogenetic analyses

The complete 5-gene data set was analyzed using ML in RAxML and Bayesian inference (BI) in MrBayes version 3.1.2 [61]; both were run on the XSEDE computing cluster [54]. Both ML and BI analyses were partitioned using one DNA partition per gene and the GTR+Γ model specified, so that model parameters were estimated independently by partition. RAxML runs were executed using the rapid ML search and bootstrapping options with 5000 replicates, and repeated several times with random starting trees to verify both topology and clade support values. MrBayes runs were started with uniform priors and consisted of four concurrent incrementally heated chains (Metropolis-coupled Markov Chain Monte Carlo, MCMC) [61], sampling every 10^3^ generations over 20^7^ generations each. Four independent runs from random starting trees (two sets of two runs each) were compared by plotting –ln likelihood per generation in Tracer v1.5 [62], and comparing marginal densities after discarding the first 10% of samples as “burn-in.” Convergent MCMC searches allowed us to combine and summarize runs in TreeAnnotator v1.5.4 [63], resulting in one maximum clade credibility tree with the best *a posteriori* topology and nodes annotated with Bayesian posterior probabilities (PP).

### Fossil calibrations

Following the best-practice recommendations of Parham et al. [64], we justified fossil calibrations with reference to the fossil taxon, locality and stratgraphic level of collection, evidence supporting the geologic age estimate, and phylogenetic analysis identifying fossil placement. We selected three fossil calibrations for these analyses; all were set as minimum dates using lognormal priors, which assumes that lineages originated no later than their oldest confidently assigned fossil member. No calibrations were constrained to be monophyletic. First, the root age of the tree, representing the most recent common ancestor (MRCA) of Chinchilloidea/Octodontoidea, was constrained using the oldest stem octodontoid, *Draconomys verai*[65,66], from the early Oligocene Sarmiento Formation at Gran Barranca, Argentina 31.1–29.5 Ma, pre-Deseadan SALMA—South American Land Mammal Age [66]. A minimum age of 29.5 Ma calibrated this node (upper 95%: 29.5–34.7 Ma, mean: 0, and standard deviation, SD: 1). Second, the MRCA of Octodontidae/Ctenomyidae was calibrated using a minimum age of 5.7 Ma (upper 95%: 5.7–10.9 Ma, mean: 0, SD: 1) to correspond to the stem ancestor of *Ctenomys*, *Xenodontomys simpsoni*[67], from the late Miocene Los Salitrales Formation at Laguna Chasicó, Argentina 6.0–5.7 Ma, late Huayquerian SALMA [67,68]. Third, the MRCA of the *Thrichomys*-*Myocastor*-*Proechimys* clade was set to a minimum of 6.0 Ma (upper 95%: 6.0-11.2 Ma, mean: 0, SD: 1) using the stem ancestor of the *Thrichomys* lineage, *Pampamys emmonsae*[69,70], from the late Miocene Cerro Azul Formation at Laguna Chillhué, Argentina 6.0-9.3 Ma, Chasicoan-Huayquerian SALMA [71].

### Divergence-time analyses

We estimated clade divergence times using the Bayesian relaxed-clock model implemented in BEAST 1.7.4 [72]. BEAST analyses were run under the GTR+Γ model with four gamma categories, unlinking site models across all five gene partitions and estimating base frequencies. Relaxed clock models were unlinked except for the two mtDNA genes (linked on the same strand) and rates were uncorrelated so that each branch was estimated from independent draws of a lognormal distribution. Clock means were set to uniform with a large upper bound. Tree models were linked and the tree prior was set to Yule, assuming a pure birth speciation process. MCMC chain lengths were set to 20^7^ generations with parameters sampled every 10^3^ generations. Four independent runs were performed on the XSEDE computing cluster and combined in order to converge upon stable posterior distributions, as determined using Tracer. Trees were summarized into a combined maximum clade credibility tree using TreeAnnotator after discarding the first 10-20% of each run as burn-in. The resulting phylogeny containing mean divergence times and error bars for each node (95% highest posterior density [HPD] intervals) was plotted in R using the ape and phyloch packages [73,74].

### Biogeographic reconstruction

To identify ancestral geographic ranges throughout the phylogeny and calculate the likelihood of different biogeographic scenarios, we used the dispersal-extinction-cladogenesis (DEC) model in Lagrange [25,75]. This ML method estimates geographic range evolution using a phylogenetic tree with branch lengths scaled to time, a set of geographic areas for all tips, and an adjacency matrix of plausibly connected areas. To focus on the species-level biogeographic history of the in-group arboreal clades, we pruned the BEAST ultrametric tree and excluded a total of thirty tips. The resulting 22-taxon tree retained single representatives for each species except *M. hispidus*, which was represented by several distinctive subclades. Instances of low nodal support (e.g., PP < 0.95) were not collapsed into polytomies because this action is prohibited when using the DEC model [25]. We coded extant species as inhabiting the Andes, Amazonia, or the Atlantic Forest, and designated the Amazon as the only connection among the regions. Ancestral range estimates were limited to at most two regions at a time. The resulting reconstructions returned all models within two likelihood units of the best model, which we parsed and summarized for each daughter branch. Relative probabilities greater than 10% were plotted along the ultrametric tree.

## Results

### Sequence characteristics

Mean base frequencies of A, C, G and T across the single-gene data sets are 0.307, 0.262, 0.125, and 0.305, respectively, for cyt-*b*; 0.374, 0.210, 0.178, and 0.235 for 12S rRNA; 0.284, 0.259, 0.232, and 0.223 for GHR; 0.213, 0.291, 0.304, and 0.187 for vWF; and 0.260, 0.260, 0.265, and 0.214 for RAG1. Tests for possible base-composition heterogeneity are not significant for any of the single-gene data sets (*P* ≈ 1.00). Significant heterogeneity in base composition for the 5-gene data set (χ^2^ = 1078.0, *P* = 0.00) appears due to proportionately fewer G’s overall (0.289, 0.258, 0.207, and 0.244) and significant phylogenetic signal P < 0.01; [76] in each data set: cyt-*b* (skewness, g_1_ = −0.557), 12S rRNA (g_1_ = −0.679), GHR (g_1_ = −1.068), vWF (g_1_ = −0.980), RAG1 (g_1_ = −1.075), and 5-gene (g_1_ = −0.536). There are 461 parsimony-informative sites in the cyt-*b* data set, 166 in 12S rRNA, 184 in GHR, 156 in vWF, and 116 in RAG1 for a total of 1083 in the 5-gene data set.

To verify aDNA results, we assembled multiple overlapping gene fragments for each gene and repetitively amplified each fragment [77]. Because instances of polymorphism among fragments could reflect either DNA damage or true heterozygosity, we coded all polymorphic sites with the corresponding IUPAC ambiguity codes. For *Toromys grandis*, we generated a longer cyt-*b* fragment (880 bp) than reported in Patterson and Velazco [37], and derived GHR and vWF fragments from a combination of amplicons from an existing DNA extract and a new extract from the same individual. All the other new aDNA sequences are from single DNA extracts as detailed in Table 1.

### Phylogenetic analyses

**Figure 3:**
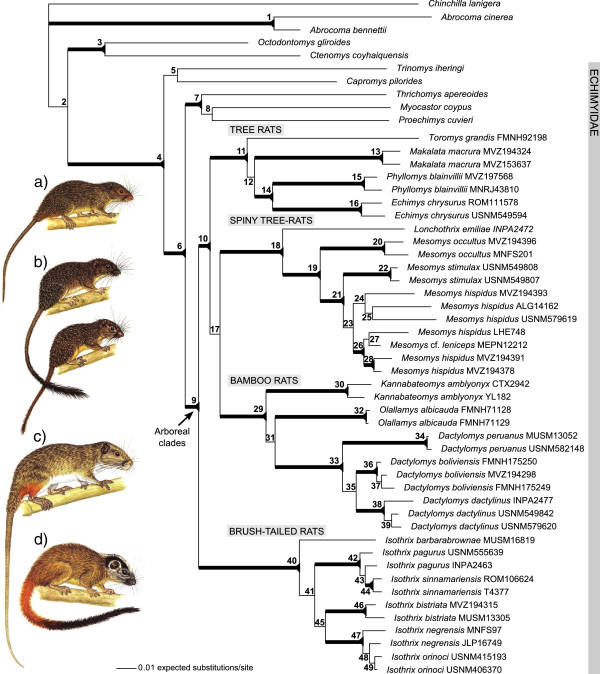
**Phylogeny of rodents in the family Echimyidae and their relatives. **The four arboreal clades are: **(a)** tree rats (pictured: *Makalata* sp.), **(b)** spiny tree-rats (pictured: *Lonchothrix emiliae* and *Mesomys stimulax*), **(c)** bamboo rats (pictured: *Dactylomys dactylinus*), **(d)** and brush-tailed rats (pictured: *Isothrix bistriata*). Evolutionary relationships were inferred from the Bayesian analysis of the combined 5-gene data set (cyt-*b* + 12S rRNA + GHR + vWF + RAG1). Numbers at nodes refer to values listed in Table 2 for Bayesian posterior probability (PP) and maximum likelihood bootstraps (ML). Thick branches indicate statistically supported relationships >0.95 PP and >75 ML. See Table 1 and Additional file 1 for details of gene sampling and specimen localities. Rodent illustrations are by Fiona A. Reid [41] and are reprinted with permission.

Node-by-node comparison of individual gene ML trees reveals no conflict across markers among statistically supported nodes (ML bootstrap support > 75; Table 2). There is variation in sister-group relationships among incompletely sampled gene trees, but the overall topological congruence allows us to confidently analyze the combined data set. The 5-gene data set yields a Bayesian posterior sample of 7.2 × 10^4^ trees after burn-in (−ln likelihood = 30,896), which converges on a single optimum as confirmed by Tracer. The best-scoring ML tree for the 5-gene data set (−ln likelihood = 30,337) is highly concordant with the BI topology. We regarded nodal support as robust with values of Bayesian PP > 0.95 and ML bootstrap support > 75 (Figure 3).

Our combined 5-gene tree (Figure 3) finds statistical support for a monophyletic Echimyidae that includes *Capromys* and *Myocastor* (node 4; Table 2). The main clade of arboreal Echimyidae is robustly recovered (node 9; Table 2; Figure 3) uniting four separate monophyletic units. Three of these units are in turn united as monophyletic (node 10; Table 2): tree rats, consisting of *Toromys, Makalata, Phyllomys + Echimys*; spiny tree-rats, consisting of *Lonchothrix* + *Mesomys*; and, bamboo rats, consisting of *Kannabateomys*, *Olallamys*, *Dactylomys*. The tree rat clade is weakly recovered as sister to the pairing of spiny tree-rats and bamboo rats (node 17; Table 2), but poor support renders this relationship an unresolved polytomy. Securely outside and sister to this polytomy are the brush-tailed rats, *Isothrix* (nodes 10 and 40; Table 2).

*Isothrix* forms a well supported monophyletic unit containing six species (node 40; Table 2; Figure 3). *I. barbarabrownae* is sister to a group that includes (*I. pagurus + I. sinnamariensis*) + (*I. bistriata* / *I. negrensis + I. orinoci*). Node 41 supports the exclusion of *I. barbarabrownae* from the rest of *Isothrix* and has strong support from Bayesian PP (1.00), but only marginal support from ML boostraps (68%). This node is well supported by cyt-*b* (ML: 91%), however it is absent in the four other gene trees, with *I. barbarabrownae* ambiguously resolved as sister taxon to *pagurus + sinnamariensis* (12S rRNA), *orinoci + bistriata* (GHR), *bistriata* (vWF), and *orinoci* (RAG1). Since each of these alternative gene histories is poorly supported (ML < 75), and hence not contradictory, results from the 5-gene phylogeny appear to best reflect this basal branching event in *Isothrix*. Other well supported groupings in the combined data set include *pagurus + sinnamariensis* (node 42) and *negrensis + orinoci* (node 47), however we do not recover strong support for the reciprocal monophyly of the species in these groupings.

**Figure 4:**
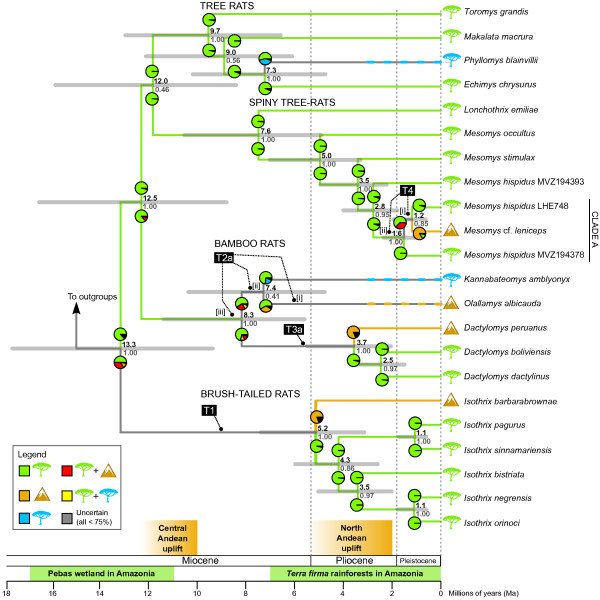
**Timetree and biogeographic reconstruction for species of arboreal spiny rats.** Geographic ranges are coded as Amazon (green tree), Andes (brown mountain), or Atlantic Forest (blue tree). Divergence time estimates at nodes are means (black) with error bars (light gray; 95% HPD) above posterior probabilities for given relationships (dark gray), and were pruned from the Bayesian relaxed clock analyses of all taxa and 5 genes (cyt-*b* + 12S rRNA + GHR + vWF + RAG1). Ancestral geographic ranges are estimated using maximum likelihood in Lagrange. Pie chart(s) represent the relative probability that the ancestors of each daughter branch occupied a given region immediately after speciation. Branches are colored to correspond with ancestral ranges of > 75% probability; gray branches are uncertain ancestral ranges, and dashed branches indicate inferred dispersal along a branch. Andes-Amazon transitions (T1–T4) are marked along the branches preceding a transition, and represent instances of either dispersal or vicariance from stem ancestors to descendant taxa (see Table 4 for additional details). Transitions T2a and T3a should be compared to the alternative topologies in Figure 5. Some transitions might have occurred along the branch of the [i] stem ancestor, [ii] two branches back, or [iii] three branches back. The timing of all geological epochs is from Gradstein et al. [78].

For the bamboo rat clade, the relative positions of all three genera are uncertain due to poor resolution at node 31 (Table 2; Figure 3). This uncertainty is also shown in the BEAST analysis with low nodal support for a re-drawn *Kannabateomys* + *Olallamys* relationship (PP: 0.41; Figure 4). We also find similar degrees of cyt-*b* divergence for *Dactylomys* from each *Olallamys* (13.4%) and *Kannabateomys* (13.5%) as the latter two share with each other (13.1%; Additional file 3). For the nuclear exon vWF (797 bp of overlapping sequence), *Dactylomys* differs by fewer substitutions from *Olallamys* (21) than from *Kannabateomys* (30) or the latter two from each other (30). However, the vWF gene tree poorly supports a *Dactylomys*-*Olallamys* relationship (49%; node 31), indicating mixed phylogenetic signal. Within a monophyletic *Dactylomys* (node 33), *D. peruanus* is sister to the pairing of *D. boliviensis* and *D. dactylinus*. Node 35 unites *D. boliviensis* + *D. dactylinus* to the exclusion of *D. peruanus*, however, with marginal support from Bayesian PP (0.85) and ML bootstraps (85%). When the analysis is restricted to the 798 bp of cyt-*b* sampled for *D. peruanus*, Bayesian support increases to 1.00 (data not shown) [37].

Within the spiny tree-rat clade, *Mesomys* (node 19) is robustly recovered, as is the basal position of *M. occultus* to the rest of the *Mesomys* radiation (node 21; Table 2; Figure 3). *M. stimulax* is sister to the *M. hispidus* clade, though with marginal support (node 23). The *M.* cf. *leniceps* specimen from the Ecuadorian Andes is nested well within *M. hispidus* specimens from the Amazonian lowlands (node 26), forming a group with individuals from northwestern Bolivia (LHE 748) and western Brazil (MVZ 194391 and MVZ 194378) as part of “clade A” identified by Patton et al. [19]. A mean cyt-*b* divergence of 3.1% separates *M.* cf. *leniceps* from other members this clade (Additional file 3). Of the six *M. hispidus* clades identified [19,42], A-D are represented here and recovered as distinctive in our phylogenetic analyses, and separate analysis of cyt-*b* sequences from all six clades confirms the affinity of *M.* cf. *leniceps* with clade A (data provided by J. L. Patton; analyses not shown). This population is known from a single specimen collected in Bosque Protector Kutukú-Shaimi (Ecuador, Prov. Morona Santiago) at 1581 m elevation. The individual was captured during a heavy rain storm after it fell from a tree. The forest had a 30 m canopy and the sample was captured in association with other small mammal species that have both Andes-restricted (*Nephelomys auriventer*) and Amazon-plus-Andes (*Marmosa lepida*, *Hylaeamys yunganus*) distributions [32]. The specimen largely agrees with the description of *Mesomys leniceps* Thomas and St. Leger 1926 [79], which was taken in Yambrasbamba (Amazonas, Peru) at 1981 m, nearly 340 km to the south. In contrast, all known records of *M. hispidus* in Ecuador lie between 200 and 980 m elevation [80]. For now, our *M.* cf. *leniceps* specimen can be safely synonomized with *M. hispidus*; however, further comparison with the holotype of *M. leniceps* will be needed before this taxon can be synonymized with *M. hispidus.*

### Divergence-time analyses

Our analysis of temporal diversification yields a BEAST posterior sample of 5.2 × 10^4^ trees after burn-in (−ln likelihood = 30,214), from which all divergence time estimates are derived. These results are depicted in Table 3, Additional file 4, and the pruned topology of Figure 4. The crown divergence of Echimyidae is estimated at 16.3 Ma with a broad error bar (95% HPD: 11.3, 21.7), followed shortly at 14.3 Ma (10.1, 19.0) by the divergence of the main arboreal clade from terrestrial echimyids. The ensuing radiation of arboreal members at 13.3 Ma (9.4, 17.5) resulted in four component clades in the following order: tree rats 9.7 Ma (6.6, 12.9), bamboo rats 8.3 Ma (5.6, 11.3), spiny tree-rats 7.6 Ma (4.9, 10.5), and brush-tailed rats 5.2 Ma (3.1, 7.4). Among Andes-Amazon distributed clades, the radiations of *Mesomys* and *Isothrix* appear approximately contemporaneous at 5.0 Ma (3.3, 7.0) and 5.2 Ma (3.1, 7.4), respectively. *Dactylomys* appeared later at 3.7 Ma (2.1, 5.5), roughly the same time as subsequent divergences between *M. stimulax* and *M. hispidus* [3.5 Ma (2.2, 4.9)] and *I. bistriata* / *I. negrensis* + *I. orinoci* [3.5 Ma (2.0, 5.0)]. However, these latter divergence intervals and those of *Mesomys* and *Isothrix* overlap by 50-70%, so none of these dates differ significantly. Comparing the temporal patterns we recover to other recent studies of caviomorph and echimyid divergence timing (Table 3), we find similar mean age estimates and degrees of error.

**Table 3:** Comparison of divergence times found in this study with values from previous studies

	**This study**	**Upham and**	**Fabre et al.**	**Galewski**	**Leite and**
		**Patterson (2012)**	**(2012)**	**et al. (2005)**	**Patton (2002)**
Chinchilloidea / Octodontoidea	30.4 (29.5, 31.9)	32.7 (30.3, 36.4)	*	*	***
Octodontoidea	22.6 (17.0, 28.3)	26.8 (24.8, 28.9)	*	*	***
Echi-Capr / Octo-Cten	20.9 (15.6, 27.0)	25.3 (24.6, 26.7)	25.1 (24.1, 26.5)	*	~11 Ma
Octo-Cten	16.3 (10.4, 22.6)	19.1 (14.3, 23.5)	20.1 (18.7, 23.2)	*	~7.5 Ma
Echi-Capr	16.0 (11.3, 21.7)	18.8 (17.7, 20.6)	18.8 (17.5, 20.2)	22.4 (14.9, 30.1)	~8 Ma
Main arboreal clade	13.3 (9.4, 17.5)	15.6 (13.9, 17.6)	15.3 (13.8, 16.7)	14.4 (8.2, 22.1)	~7 Ma
Tree rat clade	9.7 (6.6, 12.9)	11.2 (9.2, 13.5)	9.8 (8.4, 11.4)	11.5 (6.1, 18.6)	~5.5 Ma
Spiny tree-rat clade	7.6 (4.9, 10.5)	7.2 (4.7, 9.9)	8.8 (6.7, 11.9)	*	~4.5 Ma
*Mesomys*	5.0 (3.3, 7.0)	5.2 (3.1, 7.7)	**	*	**
*M. hispidus*	2.8 (1.8, 4.0)	**	***	**	*
*M.* cf. *leniceps* / *M. hispidus* LHE748	1.2 (0.6, 1.9)	***	***	***	***
Bamboo rat clade	8.3 (5.6, 11.3)	10.2 (7.0, 13.3)	9.2 (7.2, 11.5)	9.5 (4.4, 16.4)	~3.5 Ma
*Dactylomys*	3.7 (2.1, 5.5)	3.6 (1.9, 5.8)	**	**	**
rest of *Dactylomys* – *D. peruanus*	2.5 (1.5, 3.6)	***	***	***	***
Brush-tailed rat clade (*Isothrix*)	5.2 (3.1, 7.4)	4.8 (1.8, 10.3)	**	*	**
rest of *Isothrix* – *I. barbarabrownae*	4.3 (2.6, 6.0)	2.2 (0.8, 4.3)	***	***	***

Abbreviations: *Echi* Echimyidae, *Capr* Capromyidae, *Octo* Octodontidae, *Cten* Ctenomyidae.

Upham and Patterson (2012) values are from an analysis of 12S rRNA, GHR, vWF, and RAG1 for 29 echimyid species; Fabre et al. (2012) values are from their “ALL (IncludeStem)” analyses of APOB, GHR, RBP3, RAG1, vWF, cyt-*b*, 12S rRNA, and 16S rRNA for 15 echimyid species; Galewski et al. (2005) values are from their analysis of vWF amino acids for 20 echimyid species; and Leite and Patton (2002) values are from their analysis of cyt-*b*, 12S rRNA, and 16S rRNA for 14 echimyid species, using a hard minimum constraint of 7.9 ± 1.1 Ma on the *Thrichomys* lineage (by comparison, the present study used a soft minimum on the same lineage).

* Divergence time not reported in given analysis. ** Only one taxon sampled in given analysis. *** Taxon not sampled in given analysis.

All times are in millions of years and refer to estimated ages (and confidence intervals) of the specified crown groups. Results from this study are from the topology in Figure 4 and Additional file 4.

**Figure 5:**
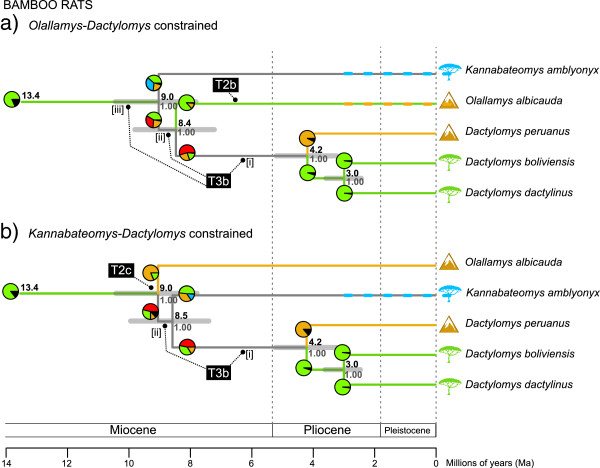
**Alternative hypotheses for the evolution of bamboo rats.** Sister relationships are constrained to be either **(a)***Olallamys*-*Dactylomys* or **(b)***Kannabateomys-Dactylomys*. These topologies are statistically equivalent to the unconstrained topology in Figure 4 that finds an *Olallamys*-*Kannabateomys* relationship and all three genera unresolved in a polytomy (0.41 PP). Each analysis is pruned from results conducted in BEAST on the full 5-gene data set. For symbols, refer to the legend in Figure 4. See Table 4 for additional details on transition timing and polarity.

Two alternative analyses were also performed constraining the monophyly of *Olallamys-Dactylomys* and *Kannabateomys-Dactylomys* (Figure 5). These runs reached stable posterior distributions with different numbers of trees (3.9 × 10^4^ and 5.2 × 10^4^ after burn-in, respectively) and resulted in identical –ln likelihood scores of 31,769. Tests developed by Shimodaira and Hasegawa [81]; phangorn package in R using 10,000 bootstrap replicates show that these three topologies for bamboo rats are not significantly different from each other (all *P* > 0.05). The two constrained topologies both find ages of 9.0 Ma (7.7, 10.3) for the bamboo rat clade and 4.2 Ma (3.2, 5.1) for the *Dactylomys* crown, and respectively find ages of 8.4 Ma (7.1, 9.7) for the *Olallamys-Dactylomys* crown, and 8.5 Ma (7.3, 9.8) for the *Kannabateomys-Dactylomys* crown (Figure 5).

### Biogeographic reconstructions

Estimating ancestral geographic ranges using Lagrange, we identify at least four transitions between the Andes and Amazon within arboreal clades of echimyid rodents (T1-T4 in Figure 4; Table 4). The ancestor to all four arboreal clades is most likely to have occupied an Amazonian range in the lineages leading to tree rats + relatives (P = 0.86) and *Isothrix* (P = 0.50), but an Andean range early in the *Isothrix* radiation cannot be excluded—either spanning both regions (P = 0.34), or the Andes alone (P = 0.13). We considered models with less than 0.75 probability to be uncertain, so without a model over this threshold, the origin of the crown *Isothrix* radiation and polarity of its biogeographic transition (T1; Figure 4) could not be determined. In analyses alternatively constraining the root for these arboreal clades, we found that an Amazon-only origin is more likely than Andean-only (global ML: -lnL = 32.06 and 36.14, respectively), but neither of these results are as likely as the mixed ancestral ranges we found in the unconstrained analysis (−lnL = 31.27; Figure 4). Hence, the most likely analysis does not exclude the possibility of an Andean or Andes + Amazon range for *Isothrix*’s stem ancestor.

**Table 4:** **Andes-Amazon transitions for echimyid rodents, as numbered in Figures**4**and**5

	**Transition**	**Branch**	**Time**		**Origin**	**Likelihood of stem ancestor’s range**
			**Earliest (stem)**	**Latest (crown)**	**(and polarity)**	
**Figure**4						
T1	*Isothrix*		13.3 (9.4, 17.5)	5.2 (3.1, 7.4)	Uncertain (? **→** A, B)	P(A): 0.50, P(AB): 0.34, P(B): 0.13
T2a	*Olallamys*	[i]	7.4 (4.8, 10.3)	Recent	Uncertain (? **→** B)	P(A): 0.58, P(B): 0.33
		[ii]	8.3 (5.6, 11.3)	7.4 (4.8, 10.3)	Uncertain (? **→** B, C)	P(A): 0.60, P(AB): 0.23, P(AC): 0.10
		[iii]	12.5 (8.8, 16.4)	8.3 (5.6, 11.3)	**Amazon (A →?)**	**P(A): 0.86**, P(AB): 0.10
T3a	*Dactylomys*		8.3 (5.6, 11.3)	3.7 (2.1, 5.5)	Uncertain (? **→** A, B)	P(A): 0.71, P(B): 0.12, P(AB): 0.11
T4	*Mesomys*	[i]	1.6 (0.9, 2.3)	1.2 (0.6, 1.9)	Uncertain (? **→** A, B)	P(A): 0.63, P(AB): 0.37
		[ii]	2.8 (1.8, 4.0)	1.6 (0.9, 2.3)	**Amazon (A → AB)**	**P(A): 0.95**
**Figure**5**a**						
T2b	*Olallamys*		8.4 (7.1, 9.7)	Recent	**Amazon (A → B)**	**P(A): 0.85**, P(B): 0.11
T3b	*Dactylomys*	[i]	8.4 (7.1, 9.7)	4.2 (3.2, 5.1)	Uncertain (? **→** A, B)	P(AB): 0.56, P(B): 0.25, P(A): 0.16
		[ii]	9.0 (7.7, 10.3)	8.4 (7.1, 9.7)	Uncertain (? **→** B, AB)	P(A): 0.40, P(AB): 0.33, P(B): 0.23
		[iii]	13.4 (11.8, 15.0)	9.0 (7.7, 10.3)	**Amazon (A → ?)**	**P(A): 0.78**
**Figure**5**b**						
T2c	*Olallamys*		13.4 (11.9, 15.0)	9.0 (7.7, 10.3)	**Amazon (A → B)**	**P(A): 0.82 → P(B): 0.77**, P(A): 0.14
T3c	*Dactylomys*	[i]	8.5 (7.3, 9.8)	4.2 (3.2, 5.1)	Uncertain (? **→** A, B)	P(AB): 0.46, P(A): 0.34, P(B): 0.17
		[ii]	9.0 (7.7, 10.3)	8.5 (7.3, 9.8)	Uncertain (? → C, AB)	P(A): 0.49, P(B): 0.36, P(C): 0.12

The earliest and latest possible times of transition are listed as the stem group and crown group divergences, respectively. Transitions that are uncertain and may have occurred on more than one branch in the given phylogeny are noted as [i] stem ancestor, [ii] two branches back, and [iii] three branches back. Likelihoods represent the probability that a stem ancestor inhabited a given region immediately after speciation. Confidently reconstructed ancestral ranges (in bold) have a likelihood greater than 0.75; otherwise, ancestral ranges and transition polarities were considered uncertain. Regions are coded as A = Amazon, B = Andes, AB = Amazon + Andes, and AC = Amazon + Atlantic Forest.

For transitions within the bamboo rat clade involving *Olallamys* (T2) and *Dactylomys* (T3), we reconstructed ancestral geographic ranges on the three possible topologies to explore how alternative sequences of diversification influence transition polarity (Figures 4 and 5). All three topologies confidently recover the stem ancestor to the bamboo rat clade as Amazonian (0.86 < P < 0.78) and thereby determine an Amazon-to-Andes polarity for at least one of the two bamboo rat transitions (Table 4). The analysis with *Kannabateomys* and *Dactylomys* as sisters (Figure 5b) confidently finds an early transition to the Andes for *Olallamys* (T2c), followed by uncertain ranges for the stem ancestors leading to *Dactylomys*. Two analyses favor an Amazon + Andes origin for *Dactylomys* (P = 0.56 and 0.46; Figures 5a and b), but none of our models recover their ancestral range with any certainty (P < 0.75; Table 4). Amazonian origins are marginally favored for *Olallamys* and *Dactylomys* in the Figure 4 topology (P = 0.58 and 0.71), and with confidence for *Olallamys* in Figure 5a (P = 0.85), but Andean ranges cannot be ruled out for *Dactylomys* in either case (Table 4). In *Mesomys*, the transition to the Andean species *M*. cf. *leniceps* (T4) is recovered as originating in the Amazon, but only with confidence for the stem ancestor of *M. hispidus* “clade A” (P = 0.95). While an Amazon + Andes distribution is not ruled out for the most immediate stem ancestor to *M*. cf. *leniceps*, this state is uncertain (P = 0.37) and less likely than a wholly Amazonian state (P = 0.63; Figure 4; Table 4).

## Discussion

### Arboreal rodent clades

The three genera distributed in both the Andes and Amazon, and their associated sister taxa, represent three of four arboreal clades known in Echimyidae. The common names of these clades—bamboo rats, spiny tree-rats, and brush-tailed rats—reflect their traditional designation as taxonomic units [35,36]. Our expanded molecular analyses confirm the monophyly of these clades (Figure 3), as well as the fourth clade of tree rats, which includes entirely lowland species from the Amazon Basin and Atlantic Forest [see also [26,27]. Each of the Andes-Amazon distributed clades are more speciose and widespread in the tropical lowlands than in the Andes, whereas the highland species are each restricted to small geographic ranges (Figure 2).

The four arboreal clades are jointly monophyletic (Figure 3), suggesting a single evolutionary shift from forest floor to forest canopy in echimyid rodents during the mid-Miocene (17.5-9.4 Ma; Figure 4). The coincident timing of Pebas wetland formation over much of Western Amazonia 17–11 Ma [16] might be linked to the colonization of arboreal niches in echimyid rodents (Figure 4) [28]; however, this dynamic system of rivers, lakes, swamps, and flood basins did not exclude small mammals with terrestrial adaptations from characteristic fossil deposits of this time period [18]. Differentiation of Echimyidae into four arboreal lineages appears to have occurred rapidly, with internode distances of less than 1 Ma in the first two branching events, and all lineages present by ~12 Ma (Figure 4). This shift to arboreality in echimyids would have preceeded the arrival of tree squirrels by 5–10 Ma [82], bringing them into contact with incumbent tree-dwelling lineages of erethizontid rodents, platyrrhine monkeys and didelphid marsupials. However, further analysis of arboreal origins in these rodents is premature, since various other echimyids (*Callistomys, Pattonomys, Diplomys,* and *Santamartamys*) show signs of arboreal adaptations [31] but are unsampled genetically.

### The role of the Andes and Amazon in biogeographic transitions

Within the radiations of echimyid clades we identified a total of four biogeographic transitions between the Andes and Amazon (T1-T4 in Figure 4). Two species from the Peruvian Andes, *Isothrix barbarabrownae* and *Dactylomys peruanus*, are each recovered as sister to larger Amazonian radiations (Figure 3), but whether these radiations originated from lowland or highland habitats is unclear. In contrast, Amazonian origins are more confidently identified for two species from the Andes of Colombia and Ecuador, *Olallamys albicauda* and *Mesomys* cf. *leniceps*. To determine the polarity of these transitions, we had to identify both a radiation’s outgroup and their ancestral geographic range (see Figure 1). We assessed the likelihood of such by considering branch length information (i.e., waiting times for speciation) [25] and regional connectivity in Lagrange (Figures 4 and 5).

For *Isothrix*, these analyses newly identify a sister clade: tree rats + spiny tree-rats + bamboo rats. This topology roots the radiation with a mix of lowland and highland taxa, without clearly suggesting the geographic range of their immediate ancestor (T1; Figure 4). The robust support we recover for *Isothrix*’s sister relationship (nodes 9, 10, and 40 in Figure 3; Table 2) is due to expanded gene sampling in the genus. It is the most resolved phylogenetic position of this genus to date cf. [26-28,37], but previous obstacles to identifying biogeographic polarity remain [37,40]. Our results indicate that *Isothrix* may have originated from a stem ancestor in either the Amazon, Andes, or Amazon + Andes (Table 4), giving rise to species in the Andes (*I. barbarabrownae*) and Amazon (the rest of *Isothrix*). The extended branch leading to *Isothrix* makes it difficult to specify the exact timing of this transition, but it must have occurred prior to the divergence of Andean and Amazonian species in the early Pliocene (5.2 Ma; Figure 4).

For bamboo rats, the polarity of transitions involving *Olallamys* and *Dactylomys* (T2 and T3 in Figure 4) are muddled because neither their outgroups nor branching order could be resolved (cf. Figure 1c). We find what is essentially a basal polytomy within bamboo rats (Figure 3; Table 2). No other molecular studies have yet included all three genera, but the morphological analysis of Carvalho and Salles [83] found *Olallamys* to be the most derived dactylomine and sister to the fossil genus *Paradelphomys*. More recent analyses [84] showed that this early Miocene fossil (Gran Barranca, Argentina) is instead a member of the extinct subfamily Adelphomyinae, and a stem ancestor to the clade of modern bamboo rats. One hypothesis for this pattern is that the lack of resolution we find among modern bamboo rats is real: generic lineages may have diversified from each other faster than mutations could accumulate along their internodes. This scenario has been suggested to explain the “star-phylogeny” observed across basal clades in Echimyidae using mitochondrial data [29,30]. We detected near-simultaneous branching at the bamboo rat crown (~1 Ma between divergences and overlapping 95% HPDs; Figure 4) and similar degrees of genetic divergence among genera (e.g., ~13% in cyt-*b*; Additional file 3), both of which support a scenario of rapid radiation. On the other hand, these results may reflect the need to analyze additional taxa and genes, particularly since we also did not find a confident outgroup for the bamboo rat clade among the taxa sampled (node 17 in Figure 3; Table 2). Two unsampled taxa, *Diplomys* and *Santamartamys*, respectively found in lowland and highland areas adjacent to *Olallamys* in the Northern Andes may be potential candidates to root the bamboo rat radiation. The increasing availability of genomic data, particularly for rare taxa and museum specimens [47,85], is expected to help resolve these evolutionary uncertainties.

To better understand how the bamboo rat topology affects our reconstructions of the *Olallamys* and *Dactylomys* transitions (T2 and T3), we compared results from three topologies that fit the molecular data equally well (Figures 4 and 5). For all trees, we found unambiguous support for an Amazonian range for the stem ancestor to bamboo rats, thus securing a lowland origin for their first transition to the Andes. However, the ranges of subsequent ancestors depend on which member of the clade is basal, and suggest two main scenarios for bamboo rat evolution. First, if *Dactylomys* is basal (Figure 4), then Amazonian ranges are probable (although uncertainly reconstructed) during the early history of the clade, and two independent Amazon-to-Andes transitions in *Olallamys* and *Dactylomys* are most likely. Second, if *Kannabateomys* or *Olallamys* are basal (Figures 5a and b), then one transition of each polarity is expected, and Andean or Andes + Amazon ranges are more likely early in the clade’s evolution. An earlier shift to the Andes raises the likelihood that the *Dactylomys* transition was a lowland recolonization leading to *D. boliviensis* and *D. dactylinus* (Figure 5a and b), but again, the origin of this radiation is as yet uncertain. An Amazonian origin for the lone Atlantic Forest taxon, *Kannabateomys*, is most likely in all analyses, perhaps using gallery forest connections to disperse through the Cerrado [86,87].

The spiny tree-rat *Mesomys* presents the clearest evidence of an Andean population (*M.* cf. *leniceps* from Ecuador) being derived from a widespread, variable lowland species (*M. hispidus*). This Amazon-to-Andes transition (T4) dates to the late Pliocene or early Pleistocene (2.8-1.2 Ma) when this form diverged from other *M. hispidus* (Figure 4; Table 4). However, since no other specimens of Ecuadorian *Mesomys* have been sampled for genetic material, the timing of highland transition for the *M.* cf. *leniceps* population should be considered tentative.

For arboreal taxa sampled in Echimyidae, we have an unequal proportion of tip data in the Amazonian state (72.7%) compared to Andean (18.2%) or Atlantic Forest (9.1%) states, so we reasonably have more statistical power for identifying transitions originating in Amazonia (T2 and T4) compared to the Andes. Given fewer living species with Andean ranges, and therefore less chance of finding Andean taxa in the outgroup and basal ingroup positions (Figure 1a), determining true Andes-to-Amazon events is expected to be more difficult. However, the topographical heterogeneity of the Andes may buffer species from local extinction, and preserve remnant highland lineages that were formerly widespread [88]. Hence, our inability to rule out Andean origins for two transitions (*Isothrix* and *Dactylomys*) is noteworthy, and establishes the reasonable possibility that Andean ranges existed early in the evolution of these lineages. If the weight of evidence eventually supports that scenario, then their speciose lowland radiations would serve as a reminder that present-day richness is not always a useful indicator of geographic origin.

### Timings of diversification in the Andes and Amazon

Considering the diversification trends in Echimyidae, are the polarities of Andean or Amazonian transitions related to their timings? Coordination between when members of a lineage transitioned between regions and the direction of their biogeographic exchange is expected if the same geological or climatic processes initiated transitions in multiple lineages. Alternatively, other idiosyncracies may be at work, such as where individual lineages originated or the ecological characteristics of species relative to environmental changes.

Geological studies now support a discrete timeline of events in the Neogene history of tropical South America [17], which can be used to examine the evolution of Echimyidae and other animal lineages. Several stages of Andean orogeny are well supported, with major uplift in the Central Andes 12–10 Ma [11] reaching a height of ~1500 m by 10 Ma, followed by at least 2300 m of additional uplift since [89,90]. Major growth of the Northern Andes was not triggered until ~5 Ma, but subsequent uplift was rapid. By 2 Ma, the full modern elevation of both the Central and Northern Andes was reached [90]. Prior to those principal orogenies, stages of Andean uplift in the Early Miocene altered drainage patterns in the Amazon Basin, creating an inland fluvial system of swamps, lakes, and some drier floodplains that encompassed most of northwest Amazonia [17,18]. Shifting drainage patterns to the east and lowered global sea levels led to the recession of this Pebas system and establishment of the east-flowing Amazon River, so that by ~7 Ma, *terra firma* rainforests had expanded widely in the Amazonian lowlands [16,17,91].

Given these geological dynamics *a priori*, we might expect to find at least three distinct types of transition between the Andes and Amazon, each confined to a specific time interval. First (type 1) is Andean lineages that are ~10 Ma or older with Amazonian roots, resulting from populations driven to colonize uplifting highland regions during wetland ingression. Adapting populations may then have been transported elevationally in step with the rising Andean Cordillera [92]. Second (type 2) is Amazonian lineages that are ~7–2 Ma with Andean roots, stemming from re-colonization of lowland *terra firma* habitats following regress of the Pebas wetland system. Andean species may be remnant ancestors to forms that radiated in novel lowland environments as the wetlands receeded [20]. Lastly (type 3) is Andean and Amazonian lineages that are ~2 Ma or younger and derived from ancestors in the other region. Populations that tracked habitats up or downslope during repeated cycles of Plio-Pleistocene climate change may have become isolated [8], so that this third type of transition might be bidirectional versus Amazon-to-Andes and Andes-to-Amazon for types 1 and 2, respectively.

Arboreal echimyids offer likely examples of these transition types as well as others. Assuming an Amazonian root for all arboreal clades at ~15 Ma (Figure 4), the first transition must have been to the Andes, perhaps in the lineage leading to *Isothrix* (13.3-5.2 Ma; Figure 4). This transition might have been a response to Central Andean uplift, implying a type 1 event, in which case we would expect a second transition of type 2 in the early Pliocene leading to the *Isothrix* lowland radiation. Alternatively, a single Andean transition leading to *I. barbarabrownae* provides a more parsimonious answer, but is not otherwise supported given current evidence. In bamboo rats, both scenarios find an Amazonian origin for *Olallamys*, but whether their ancestor transitioned to the Andes before the crown divergence of bamboo rats (9.0 Ma; Figure 5b), thus implying a type 1 event, or transitioned later along their terminal branch (8.4 Ma-Recent; Figures 4 and 5a), is uncertain. Because orogeny in the Northern Andes was not extensive until ~5 Ma [90], the simplest explanation supports a post-Pliocene arrival for the *Olallamys* lineage to its endemic range (inverse type 2 or type 3 transition). However, if proto-bamboo rats instead made an early, type 1 transition to the Central Andes, then this ancestral population may have given rise to two components: the north-dispersing ancestors of *Olallamys*, and the precursor of a highland-to-lowland radiation for *Dactylomys*. While that scenario is more complex and involves dispersal within the Andes plus a type 2 transition for *Dactylomys*, it is marginally supported depending on topology (Figure 5). For *Mesomys*, the Plio-Pleistocene (2.8-1.2 Ma) transition from the Amazon to Andes is the firmest result as it unambiguously fits the criteria for a type 3 transition. Most of the alternative biogeographic scenarios are difficult to exclude, but we expect future work will improve phylogenetic resolution for these arboreal rodent clades and yield additional insights regarding their evolution and Andes-Amazon diversification. We also expect that integrating fossil distributional data in these analyses will help illuminate geographic range shifts in echimyids, particularly since their recorded fossil history is concentrated in extra-tropical regions of southern South America.

### Reciprocal exchange among Andean and Amazonian centers of endemism

**Figure 6:**
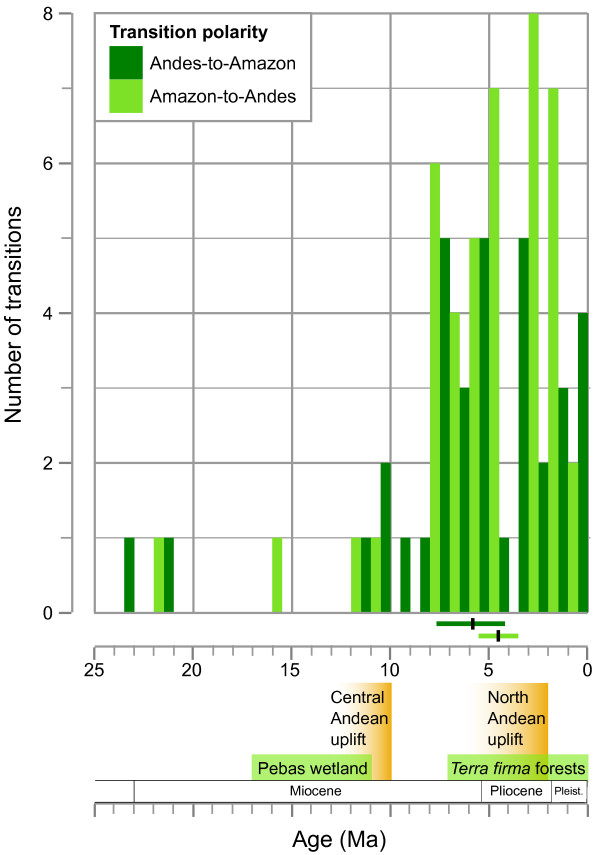
**Age and polarity of biogeographic transitions among the Andes and Amazon in other tropical lineages.** A literature search of mammal, bird, amphibian, and insect lineages finds 87 dated transitions with identifiable polarities (listed in Table 5), including 35 with origins in the Andes and 52 that originated in the Amazon. Ages of transition are grouped in 1 Ma bins and their frequency is plotted by polarity. No significant trend in timing and polarity is observed, with considerable overlap in the distribution of Andes-to-Amazon ages (dark green; mean: 5.87, 95% confidence interval [CI]: 4.12, 7.62) and Amazon-to-Andes ages (light green; mean: 4.59, 95% CI: 3.55, 5.64). We plotted midpoints for ages with date ranges, values of 0.5 Ma for ages of < 1 Ma, and did not include transitions from undated phylogenies. Relevant geologic events are shown at the bottom and additional details are provided in the Discussion.

A survey of other animal lineages in tropical South America (Table 5) offers evidence for a long-standing, and likely ongoing, exchange of species between these two megadiverse centers of endemism. Our compendium of biogeographic transitions is hardly complete, but represents an initial review of patterns in lineages with Andes-Amazon distributions, published phylogenies, and where reasonable knowledge of their geographic ranges is available. In total, we identified 87 dated transitions between these regions, with more originating in the Amazon than in the Andes (52 vs. 35; Table 5), but no significant difference in the frequency of either polarity (P > 0.05, χ^2^ = 3.32; simulated using 10,000 replicates). Transition timings range from the Early Miocene to the Middle Pleistocene (Figure 6), with all but four transitions occuring since 12 Ma, and most (88%) from 7.5 Ma onward. No significant difference exists between the mean ages of the two transition polarities (P > 0.05, t = 1.28, df = 58.3), suggesting an ongoing process of reciprocal exchange between Andes and Amazon since the end of the Miocene.

**Table 5:** Listing of animal lineages with known biogeographic transitions between Andean and Amazonian habitats

**Class**	**Group**	**Taxa**	**Highest elevation**	**Number of transitions**	**Ages of transition**	**Source(s)**
Andes-to-Amazon						
Mammals	vesper mice (Sigmodontinae)	*Calomys*	5000 m	1*	16.3% in cyt-*b* (8.2 Ma) ^a^	[21]
Mammals	olingos (Procyonidae)	*Bassaricyon*	2750 m	1*	3.5 Ma	[93]
Birds	antshrikes (Thamnophilidae)	*Thamnophilus*	2300 m	1	< 1 Ma ^b^	[14]
Birds	tanagers (Thraupidae)	*Tangara*	3500 m	6	7.5, 7.5, 5, 3, 3, and 3 Ma ^c^	[94,95]
Birds	spinetails (Furnariidae)	*Cranioleuca*	4400 m	2	1 and 0.5 Ma ^c^	[94]
Birds	miners (Furnariidae)	*Geositta*	4900 m	3	10, 9, and 5 Ma ^c^	[94]
Birds	ovenbirds (Furnariidae)	*Cinclodes*	5200 m	1	0.5 Ma ^c^	[94]
Birds	greenfinches (Fringillidae)	*Carduelis*	4600 m	2	1 and 0.5 Ma ^c^	[94]
Birds	parrots (Psittacidae)	*Pionus* (*menstruus* group)	3000 m	1*	5.6 Ma	[15]
Amphibians	poison frogs (Dendrobatidae)	*Dendrobates*	1958 m	3	21.1, 6.5, and 2.4 Ma ^d^	[20]
Amphibians	poison frogs (Dendrobatidae)	*Hyloxalus*	3500 m	5	7.4, 5.6, 5.1, 4.7, and 1.6 Ma ^d^	[20]
Amphibians	poison frogs (Dendrobatidae)	*Ameerega*	2020 m	4	7.2, 6.7, 6.1, and 3.1 Ma ^d^	[20,96]
Amphibians	salamanders (Plethodontidae)	*Bolitoglossa*	2000 m	1	23.6 Ma ^e^	[97]
Insects	butterflies (Nymphalidae)	*Ithomia*	2400 m	2	10 and 7 Ma ^f^	[98]
Insects	butterflies (Nymphalidae)	*Napeogenes*	2700 m	2	11 and 2 Ma ^f^	[98]
Amazon-to-Andes						
Mammals	spiny tree-rats (Echimyidae)	*Mesomys*	1581 m	1	2.8–1.2 Ma	This study
Mammals	bamboo rats (Echimyidae)	bamboo rat clade	3200 m	1	9.0–4.2 Ma	This study
Mammals	mouse opossums (Didelphidae)	*Marmosa* (*Micoureus*)	1634 m	1*	12.7% in cyt-*b* (6.4 Ma) ^a^	[19,99,100]
Mammals	night monkeys (Nyctipithecidae)	*Aotus*	3200 m	1	< 1 Ma ^g^	[101]
Mammals	howler monkeys (Atelidae)	*Alouatta*	3200 m	2	2.5 and 2.5 Ma ^g^	[101]
Birds	antshrikes (Thamnophilidae)	*Thamnophilus*	2300 m	2	5.5–3 and 3.6–1.6 Ma ^b^	[14]
Birds	flycatchers (Tyrannidae)	*Leptopogon*	2700 m	2	9–6 and 6–3 Ma	[12]
Birds	flycatchers (Tyrannidae)	*Myarchus*	3000 m	1	3 Ma ^c^	[94]
Birds	parrots (Psittacidae)	*Pionus* (*sordidus* group)	3000 m	1	3.0 Ma	[15]
Birds	parrots (Psittacidae)	*Amazona*	3300 m	2	2 and 1 Ma ^c^	[94]
Birds	tanagers (Thraupidae)	*Tangara*	3500 m	6	7, 4, 3.5, 3, 3, and 1 Ma ^c^	[94,95]
Birds	trogons (Trogonidae)	*Trogon*	3400 m	2	7 and 6 Ma ^c^	[94]
Birds	potoos (Nyctibiidae)	*Nyctibius*	2800 m	1	7.5 Ma ^c^	[94]
Birds	blackbirds (Icteridae)	blackbird clade	3200 m	3	5, 4, and 3.5 Ma ^c^	[94]
Birds	oropendolas (Icteridae)	*Psarocolius* and relatives	3300 m	3	5.5, 2, and 1 Ma ^c^	[94]
Birds	orioles (Icteridae)	*Icterus*	2800 m	3	7, 5.5, and 1 Ma ^c^	[94]
Birds	woodcreepers (Furnariidae)	*Xiphorhynchus*	2400 m	2	5 and 3.5 Ma ^c^	[94]
Birds	woodcreepers (Furnariidae)	*Dendrocincla*	2500 m	1	7 Ma ^c^	[94]
Birds	woodpeckers (Picidae)	*Veniliornis*	3600 m	4	4, 3.5, 1, and 1 Ma ^c^	[94]
Birds	swallows (Hirundininae)	Neotropical swallow clade	4400 m	4	11, 6, 3.5, and 2 Ma ^c^	[94]
Amphibians	poison frogs (Dendrobatidae)	*Dendrobates*	1958 m	1	4.4 Ma ^d^	[20]
Amphibians	poison frogs (Dendrobatidae)	*Ameerega*	2020 m	1	2.4 Ma ^d^	[20]
Amphibians	poison frogs (Dendrobatidae)	*Allobates*	2630 m	5	21.8, 15.2, 10.6, 1.2, and 0.8 Ma ^d^	[20]
Insects	butterflies (Riodinidae)	*Ithomiola*	2100 m	2	not dated	[13]
Insects	butterflies (Nymphalidae)	*Napeogenes*	2700 m	2	5.5 and 4.5 Ma ^f^	[98]

^a^ Divergences using cyt-*b* were converted to years using the rate 2% per Ma after Ferris et al. [102].

^b^ Age of the *T. ruficapillus* – *T. torquatus* transition was estimated from Figure 3 and the age of other splits [14].

^c^ Ages were estimated from Online Figure 2[94].

^d^ Polarity and ages of transition were obtained from Figures 2 and S12 [20].

^e^ The divergence of *B*. sp. Chilma from the remaining lowland forms Figures 3 and 5[97].

^f^ Polarities and ages were estimated from Figure 2[98].

^g^ Ages were estimated from Figure 4[101]; transitions were inferred from phylogenetic relationships and the IUCN database [32].

Polarity categories of “Andes-to-Amazon” or “Amazon-to-Andes” are based on inferences from phylogenies, geographic ranges of living taxa, and/or ancestral state reconstructions. An asterisk (*) denotes instances of outgroup uncertainty that could influence the polarity of a transition. Ages of transition are mean dates provided in the text of sources unless otherwise noted. The highest elevations for each taxon are reported from localities or databases [1,32].

Among the animal lineages surveyed, we find evidence for each of the three hypothesized types of transition, as well as transitions that do not fit our *a priori* expectations of age and polarity. Ten percent of transitions are older than 10 Ma, and only half of those meet the type 1 criteria of originating in the Amazon. Transitions from the Andes in the early and mid-Miocene are not unreasonable given that highland habitats existed in the proto-Andes and played a dynamic role in the landscape [17,91]. The additional lack of early transitions in either polarity may reflect the greater probability of extinction at this longer timescale, thereby reducing the power to reconstruct ancestral states from modern taxa alone [103]. It could also represent a real phenomenon where regional exchange among the Andes and Amazon was not common until the later Miocene. The evidence we find for 22% of transitions in the type 2 category (7.5-2.5 Ma + Andean origin) suggests that many of the initial transitions to the Andes are indeed being obscured prior to lineages recolonizing the lowlands. A large number of Amazon-to-Andes transitions also fall into the type 2 time bin (37%), and we find repeated evidence of both transition polarities after 7.5 Ma. This bidirectional pattern may indicate that widespread exchange could not begin until both regions reached their approximate modern states. Substantial highland habitats in the Andes and *terra firma* rainforests in the adjacent margins of Western Amazonia may both have been necessary for faunal exchange to develop, but were both not present until ~7 Ma [17,91]. Our expectation that type 2 transitions would originate mainly from the Andes ignored the fact that receeding Amazonian wetlands might afford greater access to eastern Andean slopes and also help foster upslope transitions. The subsequent onset of Plio-Pleistocene climate cycles appears to have encouraged reciprocal exchange, with 29% of all transitions occuring in the type 3 category (< 2.5 Ma in either polarity) and almost twice as many originating in the Amazon as the Andes (16 vs. 9).

Among terrestrial mammals (excluding bats), we found only a handful of lineages that are co-distributed in the Andes and Amazon with dated phylogenies available (Table 5). The paucity of information on mammal transitions in either polarity appears to reflect gaps in knowledge regarding species relationships and geographic ranges, particularly for small-to-medium sized mammals. One putative Andes-to-Amazon transition is observed in cricetid (sigmodontine) mice, where the genus *Calomys* contains a high-Andean clade (*C. muscilinus*, *C. lepidus*, and *C. sorellus*) as sister to a clade of wide-ranging lowland taxa [21]. While the outgroup to this pairing is as yet undetermined, recent results suggest it may be rooted with a clade of both lowland and highland taxa [104]. In this instance, a likelihood reconstruction will be necessary to tease apart biogeographic scenarios. Similarly for olingos (*Bassaricyon*), the Northern Andean species *B. neblina* is sister to a radiation of three lowland species, but rooted with both lowland and highland species in the coati genus *Nasua*[93]. Amazon-to-Andes transions are more common among the few mammal data points we gathered. For example, *Marmosa* (subgenus *Micoureus*) includes *M. regina* and *M. constantiae* with Andean distributions as sister to a clade that includes taxa in Central America, the Amazon, and Atlantic Forest (*M. alstoni*, *M. demerarae*, and *M. paraguayana*) [99,100,105]. Although this radiation’s rooting is uncertain, two wide-ranging lowland species are variously recovered as outgroups: *M. lepida*[99] and *M. murina*[105]. A simple 2% cyt-*b* per Ma conversion yields an age of 6.4 Ma for this highland transition (Table 5) [100]. Two genera of monkeys, *Aotus* and *Aloutta*, are also co-distributed in highland and lowland habitats, and each highland species likely originated in the Amazon [101].

In contrast to mammals, lineages of birds have a variety of highly resolved geographic and taxonomic data available for study [e.g., [1]. Consequently, we identified many transitions of each polarity, but over twice as many originating in the Amazon as in the Andes (37 vs. 16; Table 5). The significantly greater frequency of lowland origins in birds (P = 0.005, χ^2^ = 8.32) appears linked to events during the type 2 time bin, where there is a rate of 5.4 transitions per Ma compared to 3.6 per Ma in the type 3 bin after the Plio-Pleistocene boundary. However, the mean ages of bird transitions in each polarity show no differences, with means of 3.9 Ma (2.2, 5.7) and 4.1 Ma (3.3, 4.9) for Andean and Amazonian origins, respectively. This pattern of bidirectional exchange manifests even within several bird genera. For example, two Pliocene divergences for *Thamnophilus* antshrikes originated in the Amazon, while a third, recent shift was also made from the Andes back to the lowlands [14]. Tanagers display at least six highland-to-lowland and six lowland-to-highland transitions in an analysis of 47 species [94], with their Northern Andean populations most commonly providing a source rather than a sink for species dispersals to other zoogeographic regions [95]. *Pionus* parrots also exhibit both polarities [15]: first Andes-to-Amazon with the divergence of the species groups *menstruus* (lowland) and *chalcopterus* (highland) rooted in the Andes, and then Amazon-to-Andes with the divergence of *sordidus* (highland) and *maximiliani* (lowland) rooted in the Amazon. On the other hand, the blackbird family (Icteridae) displays nine transitions that all originated in the lowlands [94]. Flycatchers, woodpeckers, and swallows display the same unidirectional pattern [12,94], suggesting that the ecology or history of some groups favors one polarity over the other.

Amphibians show more transitions originating in the Andes than in the Amazon (17 vs. 11), but two of the poison frog genera involved, *Dendrobates* and *Ameerega*, display transitions in both directions [20,96]. Species in *Allobates* appear to have made five separate Amazon-to-Andes transitions from the early Miocene to Pleistocene, while an additional five transitions from the Andes back to the lowlands have been detected in *Hyloxalus* (Table 5) [20]. In tropical *Bolitoglossa* salamanders, at least one transition from Andean habitats occurred at the base of their mainly Amazonian radiation [97]. Insects show a balanced distribution of polarities (four vs. four) for the few cases we gathered (Table 5), including nymphalid butterflies in the genera *Napeogenes* and *Ithomia*, which display four transitions from the Andes and two from the Amazon [98].

## Conclusions

A corollary of these patterns of faunal exchange is the inference that most speciation events are actually Amazon-Amazon or Andes-Andes. Only a few rare events are trans-regional or *ex situ*, but these transitions may substantially impact the subsequent diversification of a lineage. The clades of echimyid rodents studied here are no different—although initially selected because they inhabit both the Andes and Amazon, we observe at least 14 Amazon-Amazon events compared to four transitions from one region to another (Figure 4). This majority of *in situ* transitions highlights the biome conservatism of most cladogenic events as a key feature of evolution within tropical and extra-tropical regions [106-108], particularly where topographic and hydrologic complexity restricts the movement of populations. Species are more likely to stay in the same place than to move, and more likely to retain the same ecological habits than to evolve new ones [109]. Hence, changing environmental conditions throughout Andean and Amazonian geohistory are expected to more commonly produce instances of habitat tracking and relative phenotypic stasis within a region, as compared to dispersal from another region and adaptation to a new environment. Efforts to characterize both such patterns—biogeographic exchange and stability—are therefore useful for predicting the direction of future biome shifts associated with climate change scenarios. To enable synthesis, we recommend that instances of *in situ* and *ex situ* speciation and biogeographic transition should be compiled to form a comprehensive database of plant and animal lineages. Species from this vast tropical region remain poorly known in their spatial and temporal patterns of relationship, yet contain valuable information regarding the origins, modern patterns, and future of biodiversity in tropical ecosystems.

## Availability of supporting data

The molecular data set supporting the results of this article is available in the LabArchives repository, available here: http://dx.doi.org/10.6070/H42B8VZF.

## Abbreviations

Ma: Megaannum, million years; ML: Maximum-likelihood; BI: Bayesian inference; PP: Posterior probability; MCMC: Markov Chain Monte Carlo; SALMA: South American Land Mammal Age; MRCA: Most recent common ancestor; HPD: Highest posterior density; DEC: Dispersal-extinction-cladogenesis; aDNA: Ancient DNA; mtDNA: Mitochondrial DNA.

## Competing interests

The authors declare that they have no competing interests.

## Authors’ contributions

NSU carried out the molecular genetic studies, performed all statistical analyses, helped to design the study, and drafted the manuscript. ROB and JBM collected specimens in Ecuador. PMV improved the design of the study and generated part of the molecular data. BDP conceived of the study, participated in its design and helped to draft the manuscript. All authors read and approved the final manuscript.

## Supplementary Material

Additional file 1 Collecting locality details for all in-group specimens. Click here for file

Additional file 2 Ancient DNA extraction protocol, thermal profiles, and details of PCR primers. Click here for file

Additional file 3 **Pairwise sequence divergences among taxa from the cyt-*b *data set.** Click here for file

Additional file 4 Fossil-calibrated timetree (from BEAST) for all 52 taxa in the complete 5-gene data set. Click here for file
