# Supplementary material for: Transitions between Andean and Amazonian centers of endemism in the radiation of some arboreal rodents
Source: BMC Evol Biol. 2013 Sep 9;13:191. doi: 10.1186/1471-2148-13-191 (PMC3848837; doi:10.1186/1471-2148-13-191)
Supplement: Additional file 1 — Collecting locality details for all in-group specimens. [file 1471-2148-13-191-S1.docx]

**Additional file 1** – **Upham et al. BMC Evolutionary Biology**

Collecting locality details for all in-group specimens.

| **Genus** | **species** | **Collector #** | **Museum #** | **Country** | **Prov/State** | **Dist/County** | **Specific locality** | **Lat (Dec)** | **Lon (Dec)** | **Elev (m)** |
| --- | --- | --- | --- | --- | --- | --- | --- | --- | --- | --- |
| *Makalata* | *macrura* | JLP 7197 | MVZ 153637 | PERU | Amazonas |  | Huampami (Aguaruna village), Rio Cenepa | -4.47 | -78.17 | NA |
| *Makalata* | *macrura* | JLP 15214 | MVZ 194324 | BRAZIL | Amazonas |  | near Miranda, left bank Rio Juruá | -6.75 | -70.00 | NA |
| *Toromys* | *grandis* | AMO 824 | FMNH_92198 | BRAZIL | Para |  | Rio Amazonas, Ilha de Urucurituba | -2.77 | -57.82 | NA |
| *Phyllomys* | *blainvillii* | LPC 246 | MVZ 197568 | BRAZIL | Ceará |  | Chapada do Araripe, 7 km SW Crato | -7.28 | -39.45 | 960 |
| *Phyllomys* | *blainvillii* | LMP 27 | MNRJ 43810 | BRAZIL | Minas Gerais |  | Mocambinho, Jaíba | NA | NA | NA |
| *Echimys* | *chrysurus* | LHE 555 | USNM 549594 | BRAZIL | Para |  | Altamira, 50 Km SSW, Island In Rio Xingu (Ilha Jabuti) | -3.63 | -52.37 | NA |
| *Echimys* | *chrysurus* |  | ROM 111578 | GUYANA | Potaro-Siparuni |  | Iwokrama Forest, Kabukalli | -4.28 | -58.52 | NA |
| *Dactylomys* | *boliviensis* | MNFS 988 | MVZ 194298 (INPA 2879) | BRAZIL | Acre |  | Flora [=Fazenda Santa Fé], left bank Rio Juruá | -8.60 | -72.85 | NA |
| *Dactylomys* | *boliviensis* | BDP 3942 | FMNH 175249 | PERU | Madre de Dios | Manu | Maskoitania, 13.4 km NNW Atalaya, left bank Rio Alto Madre de Dios | -12.77 | -71.39 | 480 |
| *Dactylomys* | *boliviensis* | SS 2225 | FMNH 175250 | PERU | Cusco | Paucartambo | Consuelo, 15.9 km SW Pilcopata | -13.02 | -71.49 | 1000 |
| *Dactylomys* | *dactylinus* |  | INPA 2477 | BRAZIL | Amazonas |  | Right bank Rio Purus, Município Beruri | NA | NA | NA |
| *Dactylomys* | *dactylinus* | LHE 607 | USNM 549842 | BRAZIL | Para |  | Altamira, 52 Km SSW, E Bank Rio Xingu | -3.65 | -52.37 | NA |
| *Dactylomys* | *dactylinus* | LHE 878 | USNM 579620 | BOLIVIA | Pando | Abuna Province | Ingavi, N Bank Of The Rio Orton | -10.96 | -66.83 | 150 |
| *Dactylomys* | *peruanus* | LHE 1398 | USNM 582148 | PERU | Junin |  | Satipo, Cordillera De Vilcabamba N | -11.56 | -73.64 | 2050 |
| *Dactylomys* | *peruanus* | LHE 1374 | MUSM 13052 | PERU | Junin |  | Cordillera Vilcabamba, Camp 2 | NA | NA | NA |
| *Kannabateomys* | *amblyonyx* | YL 182 |  | BRAZIL | Rio de Janeiro |  | Reserva Biologica de Poço das Antas, Silva Jardim | NA | NA | NA |
| *Kannabateomys* | *amblyonyx* | CTX 2942 |  | BRAZIL | Paraná |  | Usina Hidrelétrica Salto Caxias | NA | NA | NA |
| *Olallamys* | *albicauda* | PH 6445 | FMNH 71128 | COLOMBIA | Cundinamarca | Bogota | San Cristobal | 4.57 | -74.08 | NA |
| *Olallamys* | *albicauda* | PH 6488 | FMNH 71129 | COLOMBIA | Cundinamarca | Bogota | San Cristobal | 4.57 | -74.08 | NA |
| *Lonchothrix* | *emiliae* |  | INPA 2472 | BRAZIL | Para |  | Alter do Chão | NA | NA | NA |
| *Mesomys* | *hispidus* | MNFS 436 | MVZ 194378 (INPA 2966) | BRAZIL | Amazonas |  | Penedo, right bank Rio Jurua | -6.83 | -70.08 | NA |
| *Mesomys* | *hispidus* | MNFS 745 | MVZ 194391 (INPA 2974) | BRAZIL | Amazonas |  | Barro Vermelho, left bank Rio Juruá | -6.47 | -68.77 | NA |
| *Mesomys* | *hispidus* | LHE 748 |  | BOLIVIA | La Paz | Iturraldi | Moira Camp, Rio Madidi | -13.58 | -68.77 | NA |
| *Mesomys* | *hispidus* | LHE 836 | USNM 579619 | BOLIVIA | Pando | Abuna | San Juan De Nuevo Mundo, 18 Km N | -10.77 | -66.73 | 170 |
| *Mesomys* | *hispidus* | MNFS 909 | MVZ 194393 | BRAZIL | Amazonas |  | Altamira, right bank Rio Juruá | -6.58 | -68.90 | NA |
| *Mesomys* | *hispidus* | ALG 14162 | MBUCV | VENEZUELA | Amazonas |  | Cerro Neblina, Base Camp | NA | NA | 140 |
| *Mesomys* | cf*. leniceps* | JBM 368 | MEPN 12212 | ECUADOR | Morona Santiago |  | Yapit (antiguo camino shuar Logroño-Yaupi), Bosque Protector Kutukú-Shaimi, parroquia Shimpis, cantón Logroño | -2.72 | -78.09 | 1581 |
| *Mesomys* | *occultus* | JUR 501 | MVZ 194396 (INPA 2690) | BRAZIL | Amazonas |  | Colocação Vira-Volta, left bank Rio Juruá on Igarapé Arabidi, affluent of Paraná Breu | -3.28 | -66.23 | NA |
| *Mesomys* | *occultus* | MNFS 201 |  | BRAZIL | Amazonas |  | Upper Rio Uruco | -4.85 | -65.27 | NA |
| *Mesomys* | *stimulax* | MDC 550 | USNM 549807 | BRAZIL | Para |  | Altamira, 52 Km SSW, E Bank Rio Xingu | -3.65 | -52.37 | NA |
| *Mesomys* | *stimulax* | LHE 572 | USNM 549808 | BRAZIL | Para |  | Altamira, 52 Km SSW, E Bank Rio Xingu | -3.65 | -52.37 | NA |
| *Isothrix* | *barbarabrownae* | BDP 3878 | FMNH 170722 (MUSM16819) | PERU | Cusco | Paucartambo | Suecia, km 138.5 Carretera Shintuya | -13.10 | -71.57 | 1920 |
| *Isothrix* | *bistriata* | MNFS 471 | MVZ 194315 (INPA 2095) | BRAZIL | Amazonas |  | Penedo, right bank Rio Juruá | -6.83 | -70.08 | NA |
| *Isothrix* | *bistriata* | RSV 2293 | MUSM 13305 | PERU | Loreto |  | Nuevo San Juan, Río Gálvez | NA | NA | NA |
| *Isothrix* | *negrensis* | MNFS 97 | INPA | BRAZIL | Amazonas |  | Upper Rio Uruco | -4.85 | -65.27 | NA |
| *Isothrix* | *negrensis* | JLP 16749 | INPA | BRAZIL | Amazonas |  | right bank Rio Jaú above mouth | NA | NA | NA |
| *Isothrix* | *orinoci* |  | USNM 406370 | VENEZUELA | Amazonas |  | Boca Mavaca, 84 Km SSE Esmeralda, Rio Manavichi | 2.55 | -65.03 | 138 |
| *Isothrix* | *orinoci* |  | USNM 415193 | VENEZUELA | Amazonas |  | Capibara, 106 Km SW Esmeralda, Brazo Casiquiare | 2.62 | -66.32 | 130 |
| *Isothrix* | *pagurus* | LHE 141 | USNM 555639 | BRAZIL | Amazonas |  | Fazenda Esteio | -2.42 | -59.83 | NA |
| *Isothrix* | *pagurus* |  | INPA 2463 | BRAZIL | Amazonas |  | UHE Pitinga, Rio Pitinga (hidroelectrico) | NA | NA | NA |
| *Isothrix* | *sinnamariensis* |  | ROM 106624 | GUYANA | U Tatutu-U Essequibo |  | Chradikar R. 55 km SW Gunn's strip | NA | NA | NA |
| *Isothrix* | *sinnamariensis* |  | T4377 | FRENCH GUIANA | Régina |  | Les Nouragues | NA | NA | NA |
