# Supplementary material for: Transitions between Andean and Amazonian centers of endemism in the radiation of some arboreal rodents
Source: BMC Evol Biol. 2013 Sep 9;13:191. doi: 10.1186/1471-2148-13-191 (PMC3848837; doi:10.1186/1471-2148-13-191)
Supplement: Additional file 2 — Ancient DNA extraction protocol, thermal profiles, and details of PCR primers. [file 1471-2148-13-191-S2.docx]

**Additional file 2** – **Upham et al. BMC Evolutionary Biology**

Ancient DNA extraction protocol, thermal profiles, and details of PCR primers.

Approximately 25 mg of each sample along with one extraction blank were incubated in 0.5 ml of 0.5 M EDTA pH 8.0 (EMD Chemicals) for 24 hours at 25ºC using gentle agitation (1000 rpm). Samples were centrifuged at high speed and the EDTA supernatants were transferred to 5 ml tubes and processed separately. To the remaining tissue in each sample, we added 0.5 ml of digestion buffer [20 mM Tris (EMD Chemicals) at pH 8.0, 0.5% N-Lauroylsarcosine (Sigma), 250 mg/ml Proteinase K (Thermo Fisher Scientific), 5 mM CaCl_2_ (EMD Chemicals), 50 mM DTT (EMD Chemicals), 1% PVP (EMD Chemicals), 2.5 mM PTB (Prime Organics)]. After incubating at 55°C for 12-24 hours on a rotary wheel, samples were again spun down and the supernatant transferred to the same 5 ml tube. This dimineralization-digestion process was repeated three times and the resultant ~3 ml of supernatant formed the raw DNA extract for further purification. Leftover tissue pellets were discarded or saved for future use. Organic purification was performed on each supernatant sample, ﬁrst using 1.0 ml of phenol-chloroform-isoamylalcohol (25:24:1, pH 8, Fluka) and then 1.0 ml of chloroform (Fluka), retaining only the aqueous phase in each step. Samples were concentrated by ultrafiltration with Amicon Ultra 30K columns (Millipore), washed with three steps of 450 μl of 0.1x TE+Tween [10 mM Tris, 0.1 mM EDTA, 0.05% Tween-20 (Sigma), pH 8.0], and the DNA was finally eluted in 80 µL of 0.1x TE+Tween. PCRs performed on aDNA samples differed from fresh DNA reactions in the concentration of dNTPs (400 μM) and DNA polymerase (0.2 U), the targeting of smaller gene fragments (200-400 bp), and the inclusion of DNA extraction blanks.

PCR profiles were as follows: initial denaturation at 95°C for 5 min, followed by 35 cycles denaturation at 95°C for 1 min, annealing at 49-52°C (cyt-*b* and 12S rRNA) or 56-58°C (vWF and RAG1) for 1 min, extension at 72°C for 90 sec, and a final extension at 72°C for 10 min. GHR was amplified using the touchdown profile described by Rowe and Honeycutt [[1](#_ENREF_1)]. PCR products were purified using ExoSAP-IT (Affymetrix) and cycle-sequenced using 1.0 μl of ABI PRISM Big Dye version 3.1 (Applied Biosystems), 3.0 μl of dilution buffer, 0.4 μl of one amplification primer, 4.6 μl of dH_2_O, and 1.0 μl of PCR product. The cycling profile involved an initial denaturation at 96°C for 1 min, and 25 cycles of denaturation at 96°C for 10 sec, annealing at 50°C for 5 sec, and extension at 60°C for 4 min. Cycle-sequencing products were purified through an EtOH–EDTA precipitation protocol and run on ABI PRISM 3730 Genetic Analyzers (Applied Biosystems), either at the Field Museum, McMaster University, or Macrogen Korea (<https://dna.macrogen.com/>).

**Table A1** – Summary of PCR primers, primer pairs, and fragment lengths

| **Cytb** |  |  |  |  |  |
| --- | --- | --- | --- | --- | --- |
| **Primer ID** | **Position in gene** | **Published name** | **Source** | **Sequence 5' to 3'** | **Notes** |
| cytb_1L | 0F | MVZ05 | Smith and Patton (1993) | CGAAGCTTGATATGAAAAACCATCGTTG | position 14115 in *Mus* |
| cytb_2L | 412F | MVZ45 | Smith and Patton (1993) | ACNACHATAGCNACAGCATTCGTAGG | position 14527 in *Mus* |
| cytb_3H | 427R | MVZ04 | Smith and Patton (1993) | GCAGCCCCTCAGAATGATATTTGTCCTC | position 14542 in *Mus* |
| cytb_4L | 439F | MVZ127 | Leite and Patton (2002) | TRYTACCATGAGGACAAATATC | position 14554 in *Mus* |
| cytb_5H | 825R | MVZ16 | Smith and Patton (1993) | AAATAGGAARTATCAYTCTGGTTTRAT | position 14940 in *Mus* |
| cytb_6H | 1177R | MVZ108 | Leite and Patton (2002) | CCAATGTAATTTTTATAC | position 15292 in *Mus* |
| cytb_XL | ~0F | L14724 | Irwin et al. (1991) | CGAAGCTTGATATGAAAAACCATCGTTG | position 14724 in *Homo* |
| cytb_XH | 1189R | H15915 | Irwin et al. (1991) | AACTGCAGTCATCTCCGGTTTACAAGAC | position 15915 in *Homo* |
|  |  |  |  |  |  |
| **Fragment ID** | **Primer combo (ID)** | **Fragment length** | **bp spanned** | **Primer combo (published)** |  |
| A | 1L/3H | 427 bp | 0-427 | MVZ05–MVZ04 |  |
| B | 1L/5H | 825 bp | 0-825 | MVZ05–MVZ16 |  |
| C | 2L/5H | 413 bp | 412-825 | MVZ45–MVZ16 |  |
| D | 4L/5H | 386 bp | 439-825 | MVZ127–MVZ16 |  |
| E | 4L/6H | 738 bp | 439-1177 | MVZ127–MVZ108 |  |
| X | XL/XH | ~1140 bp | 0-1189 | L14724–H15915 |  |
|  |  |  |  |  |  |
|  |  |  |  |  |  |
| **12S** |  |  |  |  |  |
| **Primer ID** | **Position in gene** | **Published name** | **Source** | **Sequence 5' to 3'** | **Notes** |
| 12S_1L | 1F | --- | This study | CATAGACACA**A**AGGTTTGGTCC | Modified from L82N of Nedbal et al. (1994) |
| 12S_2H | 883R | Hend | Nedbal et al. (1994) | CCAAGCACACTTTCCAGTATGC |  |
| 12S_4H | internal for sequen. | H900 | Nedbal et al. (1994) | TGACTGCAGAGGGTGACGGGCGGTGTGT |  |
| 12S_5L | internal for sequen. | L309 | Nedbal et al. (1994) | GTTGGTAAATCTCGTGC |  |
| 12S_12L | 0F | MVZ59F | Leite and Patton (2002) | ATAGCACTGAAAAYGCTDAGATG |  |
| 12S_13H | 595R | --- | This study | TT**AT**AGAACAGGCTCCTCTAG | Modified from MVZ44R of Leite and Patton (2002) |
|  |  |  |  |  |  |
| **Fragment ID** | **Primer combo (ID)** | **Fragment length** | **bp spanned** | **Primer combo (published)** |  |
| A | 1L/2H | 883 bp | 0-883 | --- |  |
| seq | 4H/5L (use with A) | for sequencing | --- | L309–H900 |  |
| D | 12L/13H | 549 bp | 0-595 | --- |  |
|  |  |  |  |  |  |
|  |  |  |  |  |  |
| **GHR** |  |  |  |  |  |
| **Primer ID** | **Position in gene** | **Published name** | **Source** | **Sequence 5' to 3'** | **Notes** |
| GHR_2F | 0F | GHR50F | Adkins et al. (2001) | TTCTAYARYGATGACTCYTGGGT |  |
| GHR_3R | 891R | GHREND | Adkins et al. (2001) | CTACTGCATGATTTTGTTCAGTTGGTCTGTGCTCAC |  |
| GHR_4F | internal for sequen. | GHR10 | Rowe and Honeycutt (2002) | ACCAGCAGGNAGTGTRGTCCTTTC |  |
| GHR_5R | internal for sequen. | GHRendC | Rowe and Honeycutt (2002) | RTGGCTTACTTGGGCATAAAAGTC |  |
| GHR_10F | 47F | --- | This study | GGGTTGAATTTATTGAGCTAGATATTG | Designed from Echimyidae and *Capromys* sequences |
| GHR_11F | 277F | --- | This study | GGAGAAGCAGATCTCTTGTGCCTTG | Designed from Echimyidae and *Capromys* sequences |
| GHR_12R | 416R | --- | This study | GACTCAGTTTTACCAATAAAAAGTAGTTGTGG | Designed from Echimyidae and *Capromys* sequences |
| GHR_13F | 462F | --- | This study | GGCAAACATGGACTTTTATGCTCAAGTAAGC | Designed from Echimyidae and *Capromys* sequences |
| GHR_14R | 495R | --- | This study | GTCGCTTACTTGAGCATAAAAGTCC | Designed from Echimyidae and *Capromys* sequences |
| GHR_15R | 796R | --- | This study | CAGAGGTATAATCTGGGAGGGCCATTTC | Designed from Echimyidae and *Capromys* sequences |
|  |  |  |  |  |  |
| **Fragment ID** | **Primer combo (ID)** | **Fragment length** | **bp spanned** | **Primer combo (published)** |  |
| A | 2F/3R | 891 bp | 0-891 | GHR50F–GHREND |  |
| seq | 4F/5R (use with A) | for sequencing | --- | GHR10–GHRendC |  |
| B | 10F/12R | 369 bp | 47-416 | --- |  |
| C | 11F/14R | 218 bp | 277-495 | --- |  |
| D | 13F/15R | 334 bp | 462-796 | --- |  |
| E | 10F/14R | 448 bp | 47-495 | --- |  |
| F | 11F/15R | 519 bp | 277-796 | --- |  |
|  |  |  |  |  |  |
|  |  |  |  |  |  |
| **vWF** |  |  |  |  |  |
| **Primer ID** | **Position in gene** | **Published name** | **Source** | **Sequence 5' to 3'** | **Notes** |
| vWF_1F | 44F | V10 | Galewski et al. (2005) | TTAGTGCTACCACCCCATACCTGGAAG |  |
| vWF_2R | 878R | W2 | Huchon et al. (1999) | ACGTCCATGCGCTGGATCACCT |  |
| vWF_3R | 542R | W13 | Galewski et al. (2005) | GGCCCGATGCCCACTGGCATCA |  |
| vWF_4F | 304F | V2 | Huchon et al. (1999) | CCCTCAGAGCTGCGGCGCAT |  |
| vWF_5R | 1258R | W1 | Huchon et al. (1999) | TGCAGGACCAGGTCAGGAGCCTCTC |  |
|  |  |  |  |  |  |
| **Fragment ID** | **Primer combo (ID)** | **Fragment length** | **bp spanned** | **Primer combo (published)** |  |
| A | 1F/2R | 834 bp | 44-878 | V10–W2 |  |
| B | 1F/3R | 498 bp | 44-542 | V10–W13 |  |
| C | 4F/5R | 954 bp | 304-1258 | V2–W1 |  |
| D | 4F/3R | 238 bp | 304-542 | V2–W13 |  |
| E | 4F/2R | 574 bp | 304-878 | V2–W2 |  |
|  |  |  |  |  |  |
|  |  |  |  |  |  |
| **RAG1** |  |  |  |  |  |
| **Primer ID** | **Position in gene** | **Published name** | **Source** | **Sequence 5' to 3'** | **Notes** |
| RAG1_1F | 0F | RAG1F1705 | Teeling et al. (2000) | GCTTTGATGGACATGGAAGAAGACAT |  |
| RAG1_3F | 260F | --- | This study | GACTGCCATCCTCAGCCCGC | Designed from Echimyidae alignment |
| RAG1_4R | 1013R | --- | This study | AAGCTCGGCGAAACGCTGGG | Designed from Echimyidae alignment |
| RAG1_6R | 706R | FMNH2b | Patterson and Velazco (2008) | TTATACACCTCCCCTATCTCKAGC |  |
| RAG1_7F | 651F | FMNH3a | Patterson and Velazco (2008) | GGCAATGCHGCYGAATTCTACAAGAT |  |
| RAG1_8R | 1062R | RAG1R2864 | Teeling et al. (2000) | GAGCCATCCCTCTCAATAATTTCAGG |  |
|  |  |  |  |  |  |
| **Fragment ID** | **Primer combo (ID)** | **Fragment length** | **bp spanned** | **Primer combo (published)** |  |
| B | 3F/4R | 753 bp | 260-1013 | --- |  |
| C | 1F/6R | 706 bp | 0-706 | RAG1F1705–FMNH2b |  |
| D | 7F/8R | 413 bp | 651-1062 | FMNH3a–RAG1R2864 |  |

**References [**[**1-10**](#_ENREF_1)**]**

1. Rowe DL, Honeycutt RL: **Phylogenetic relationships, ecological correlates, and molecular evolution within the Cavioidea (Mammalia, Rodentia)**. *Mol Biol Evol* 2002, **19**:263–277.

2. Galewski T, Mauffrey J-F, Leite YLR, Patton JL, Douzery EJP: **Ecomorphological diversification among South American spiny rats (Rodentia; Echimyidae): a phylogenetic and chronological approach**. *Molecular Phylogen and Evolution* 2005, **34**:601–615.

3. Leite YLR, Patton JL: **Evolution of South American spiny rats (Rodentia, Echimyidae): the star-phylogeny hypothesis revisited**. *Mol Phylogenet Evol* 2002, **25**:455-464.

4. Patterson BD, Velazco PM: **Phylogeny of the rodent genus *Isothrix* (Hystricognathi, Echimyidae) and its diversification in Amazonia and the Eastern Andes**. *Journal of Mammalian Evolution* 2008, **15**:181-201.

5. Adkins RM, Gelke EL, Rowe D, Honeycutt RL: **Molecular phylogeny and divergence time estimates for major rodent groups: evidence from multiple genes**. *Mol Biol Evol* 2001, **18**:777–791.

6. Huchon D, Catzeflis FM, Douzery EJP: **Molecular evolution of the nuclear von Willebrand Factor gene in mammals and the phylogeny of rodents**. *Mol Biol Evol* 1999, **16**:577–589.

7. Irwin DM, Kocher TD, Wilson AC: **Evolution of the cytochrome b gene of mammals**. *J Mol Evol* 1991, **32**:128-144.

8. Nedbal MA, Allard MW, Honeycutt RL: **Molecular systematics of hystricognath rodents: evidence from the mitochondrial 12S rRNA gene**. *Mol Phylogenet Evol* 1994, **3**:206–220.

9. Teeling EC, Scally M, Kao DJ, Romagnoli ML, Springer MS, Stanhope MJ: **Molecular evidence regarding the origin of echolocation and flight in bats**. *Nature* 2000, **403**:188–192.

10. Smith MF, Patton JL: **The diversification of South American murid rodents: Evidence from mitochondrial DNA sequence data for the akodontine tribe**. *Biol J Linn Soc* 1993, **50**:149-177.
