# Supplementary material for: Transitions between Andean and Amazonian centers of endemism in the radiation of some arboreal rodents
Source: BMC Evol Biol. 2013 Sep 9;13:191. doi: 10.1186/1471-2148-13-191 (PMC3848837; doi:10.1186/1471-2148-13-191)
Supplement: Additional file 3 — Pairwise sequence divergences among taxa from the cyt-b data set. [file 1471-2148-13-191-S3.docx]

**Additional file 3 – Upham et al. BMC Evolutionary Biology**

**Table** - Pairwise sequence divergences among taxa from the cyt-*b* data set.

Pairwise divergences are calculated as means among groups of taxa. Uncorrected-p distances are reported, which summarize the raw number of substitution differences among taxa divided by their sequence lengths. Values in parentheses exclude the third codon position, which was the most variable site (321 of 461 parsimony informative characters).

Spiny tree-rats Bamboo rats Brush-tailed rats Tree rats

Spiny tree-rats *9.3% (2.3%)*

Bamboo rats 17.3% (5.6%) *10.3% (3.3%)*

Brush-tailed rats 19.3% (7.3%) 17.6% (6.8%) *9.5% (3.5%)*

Tree rats 18.2% (6.6%) 16.9% (6.3%) 18.1% (7.2%) *14.9% (5.5%)*

**Mean between clades 18.3% (6.5%) 17.3% (6.2%) 18.3% (7.1%) 17.7% (6.7%)**

**Spiny tree-rats**

*Mesomys* / *Lonchothrix* 14.8% (4.5%)

within *Mesomys* 8.2% (1.9%)

*M*. cf. *leniceps* / rest of clade A 3.1% (0.8%)

**Bamboo rats**

*Dactylomys* / *Kannabateomys* 13.5% (3.8%)

*Dactylomys* / *Olallamys* 13.4% (5.1%)

*Olallamys* / *Kannabateomys* 13.1% (4.0%)

within *Dactylomys* 6.8% (1.9%)

*D. peruanus* / rest of *Dactylomys* 9.2% (3.3%)

**Brush-tailed rats**

*I. barbarabrownae* / rest of *Isothrix* 14.8% (6.0%)

(*I. pagurus + I. sinnamariensis*) /

(*I. bistriata* (*I. negrensis + I. orinoci*)) 10.6% (3.9%)

**Tree rats**

*Toromys* / rest of tree rats 18.0% (7.0%)

*Makalata* / (*Phyllomys + Echimys*) 17.2% (5.7%)

*Phyllomys* / *Echimys* 15.0% (5.0%)

**Figure** – Nucleotide site saturation by codon position for the cyt-*b* data set of 52 taxa.
