# Supplementary figures and images for: Transitions between Andean and Amazonian centers of endemism in the radiation of some arboreal rodents
Source: BMC Evol Biol. 2013 Sep 9;13:191. doi: 10.1186/1471-2148-13-191 (PMC3848837; doi:10.1186/1471-2148-13-191)

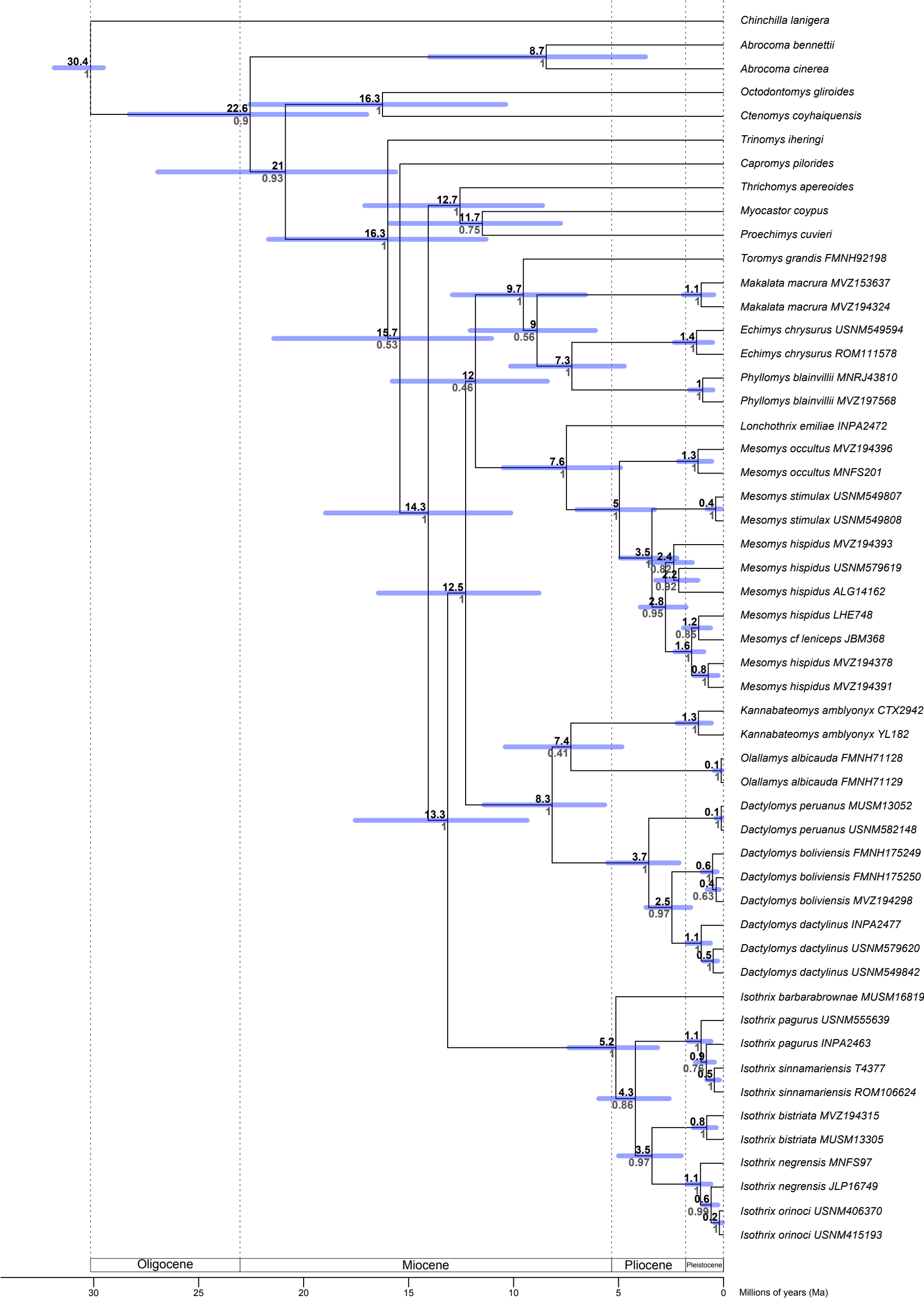

Supplement: Additional file 4 — Fossil-calibrated timetree (from BEAST) for all 52 taxa in the complete 5-gene data set. [file 1471-2148-13-191-S4.pdf]
